# Hiding in Plain Sight: Formation and Function of Stress Granules During Microbial Infection of Mammalian Cells

**DOI:** 10.3389/fmolb.2021.647884

**Published:** 2021-04-29

**Authors:** Alistair Tweedie, Tracy Nissan

**Affiliations:** ^1^Department of Biochemistry and Biomedicine, School of Life Sciences, University of Sussex, Brighton, United Kingdom; ^2^Department of Molecular Biosciences, The Wenner-Gren Institute, Stockholm University, Stockholm, Sweden

**Keywords:** stress granules (SG), eIF2 alpha, integrated stress response (ISR), unfolded protein response (UPR), PKR, PERK, GCN2, HRI

## Abstract

Stress granule (SG) formation is a host cell response to stress-induced translational repression. SGs assemble with RNA-binding proteins and translationally silent mRNA. SGs have been demonstrated to be both inhibitory to viruses, as well as being subverted for viral roles. In contrast, the function of SGs during non-viral microbial infections remains largely unexplored. A handful of microbial infections have been shown to result in host SG assembly. Nevertheless, a large body of evidence suggests SG formation in hosts is a widespread response to microbial infection. Diverse stresses caused by microbes and their products can activate the integrated stress response in order to inhibit translation initiation through phosphorylation of the eukaryotic translation initiation factor 2α (eIF2α). This translational response in other contexts results in SG assembly, suggesting that SG assembly can be a general phenomenon during microbial infection. This review explores evidence for host SG formation in response to bacterial, fungal, and protozoan infection and potential functions of SGs in the host and for adaptations of the pathogen.

## Introduction

Stress granules (SGs) are cytoplasmic structures that accumulate as foci in response to multiple cellular stresses. SGs generally assemble in response to these stresses through stress-induced inhibition of translation initiation. In mammals, this is commonly accomplished by activation of kinases that phosphorylate eukaryotic translation initiation factor 2α (eIF2α). This results in stalled 48S translation initiation complexes on mRNAs and a reduction in translating ribosomes due to further elongation without initiation. These mRNAs, cleared of ribosomes, bind to RNA binding proteins, which facilitate the process of SG assembly. Over time, assembled SGs can gain altered properties as well as recruit many additional mRNAs and proteins.

Central to the assembly of SGs, are the RNA binding proteins TIA1, TIAR, and G3BP1, which oligomerize on non-translating mRNA present in the granules. These, and other proteins, have multiple low-affinity interactions with both RNA and proteins, of which G3BP1 appears to be the central essential factor (Guillén-Boixet et al., 2020; Sanders et al., 2020; Yang et al., 2020; Hofmann et al., 2021). The proteins involved often contain intrinsically disordered regions and/or low complexity prion-like repeat sequences. These weak dynamic interactions act together to undergo the process of liquid–liquid phase separation (LLPS). This process concentrates proteins and RNA within the SGs to create a distinct fluid environment.

In infection biology, viruses were found to assemble SGs in response to viral infections soon after the discovery of SGs. Sensors for cellular stresses, such as the presence of endoplasmic reticulum (ER) stress, dsRNA, or amino acid starvation activate eIF2α kinases. Viruses were recognized early as potential activators as they often contain dsRNA or cause ER stress, commonly from viral protein production (Anderson and Kedersha, 2002).

Translation inhibition that results in SG assembly is an important response of the cell to restrict viral protein production and replication. A clue to the importance of SGs is the multiple ways in which viruses affect them (White and Lloyd, 2012). Manipulation of SGs by viruses can include eIF2α-independent SG assembly, inhibition of SG assembly (either by other stresses or by viruses), or even subversion of SG proteins for new functions and novel non-SG-like aggregates, which are used for viral purposes, including viral replication.

More recent evidence suggests that SGs function in innate immunity in combatting viruses, independently of the role of translation inhibition (Lloyd, 2012; Onomoto et al., 2014; McCormick and Khaperskyy, 2017; Eiermann et al., 2020). These functions are primarily linked to sequestration of proteins and RNA within SGs, including translation initiation factors, RNA binding proteins involved in viral replication and signaling molecules. Beyond affecting translation, the sequestration inhibits viral replication and cellular apoptosis. Further evidence of the importance of SGs in combatting viral infections is that many viruses show increased viral production without SGs. Conversely, in other cases in which SG assembly cannot be subverted by viruses, there is a reduction in viral infection due to SGs.

In contrast to the well-studied effects of viruses on SGs, the role of bacterial, fungal, and protozoan infection in mammalian cells has remained largely unexplored. We argue in this review that there exists substantial experimental evidence that microbial infection is likely to result in host SG assembly. In addition, existing evidence suggests that specific microbial mechanisms to prevent or subvert SG assembly or proteins can exist, which are likely to be important for infection.

## Modulation of Stress Granules in Host Cells as a Response to Microbial Infection

Signaling upstream of SG assembly in host cells can be activated by non-viral microbial infection. This has been studied in a number of microorganisms. In contrast, the resulting effect on SGs remains largely unexamined (Table 1). Essentially, the presence or effect of microbial infection on host cell SG formation has only been examined in a handful of organisms. These include the Gram-negative *Salmonella* and *Shigella* bacteria, the Gram-positive bacteria *Listeria*, and the protozoan parasite *Plasmodium*.

**TABLE 1:** Microbes that induce stress granule (SG) formation.

Microbe	Marker	Time	Notes	References
***L. monocytogenes***	TIA1	1 h	10% to 20% (4–5 hpi)	Abdel-Nour et al. (2019)
***S. typhimurium***	TIA1	0.5 h	2% at 0.5 hpi, increase to 5% at 5 hpi	Abdel-Nour et al. (2019)
***S. typhimurium***	TIA1	1 h	Transient (only 1–2 hpi)	Tattoli et al. (2012)
***S. flexneri***	TIA1	0.5 h	5%, to 20–30% at 3–5 hpi	Abdel-Nour et al. (2019)
***S. flexneri***	TIA1	2 h	Significant at 2 hpi; Persists until 4 hpi	Tattoli et al. (2012)
***S. flexneri***	eIF3b	2 h	Invasive *Shigella* only to about 20% at 2 hpi	Vonaesch et al. (2016)

The first microbes demonstrated to cause host SG formation were the bacteria *Salmonella typhimurium* and *Shigella flexneri* (Tattoli et al., 2012; Vonaesch et al., 2016; Abdel-Nour et al., 2019). These were identified with SG marker proteins including TIA1 and another SG component, the translation initiation factor eIF3b.

*Salmonella* infection resulted in a transient induction of TIA1^+^ SGs at 1–2 h post infection (hpi) (Tattoli et al., 2012). A later study quantitated the percentage of *Salmonella-*infected MEF cells containing SGs, finding it to be relatively low (Abdel-Nour et al., 2019). The percentage of SGs increased over time from 2% at 30 min to 5% at 5 hpi. The SGs were phosphorylated eIF2α (P-eIF2α) dependent, as they were not observed in the non-phosphorylatable eIF2α mutant (S51A) knock-in.

*Shigella* infection presents an interesting case of host SG modulation. *Shigella-*infected cells exhibited a more robust host SG response beginning with 5% of the cells exhibiting TIA1^+^ SGs at 30 min, increasing to 30% SGs at 5 hpi, which were dependent on P-eIF2α for assembly. A similar result was reported by another group using HeLa cells (Vonaesch et al., 2016). In this study, around 5% of the cells had eIF3b^+^ SGs, while non-invasive *Shigella* lacking the virulence plasmid did not exhibit any SG formation. However, *Shigella* infection was associated with a reduction in drug-induced SGs, dependent on the presence of the virulence plasmid. This was further examined by pre-infection and subsequent exposure to the drugs for 1 h (either clotrimazole or pateamine A to induce SG assembly by different mechanisms). The inhibitory effect on SGs could be seen with as little as 15 min pre-infection. The decrease in SGs observed became more pronounced over time, up to at least 2 h, which was the maximum time examined. Intriguingly, *Shigella* infection alone and in combination with SG-inducing drugs, still exhibited elevated P-eIF2α. They further examined several *Shigella* mutants, but were not able to find any that rescued the disruption of SGs. These results suggest that while the virulent wild type *Shigella* induces SG formation to a limited extent, it, nevertheless, exhibits a mechanism to inhibit SG formation despite elevated P-eIF2α.

Infection of MEF cells with *Listeria* resulted in an induction of host SGs, which was P-eIF2α dependent at 1 hpi (Abdel-Nour et al., 2019). However, the level was not sustained at later time points, but, nevertheless, rose again to higher levels at 4–5 hpi. This is suggestive of a possibility of SG oscillation in bacterial cells as has been previously observed during hepatitis C virus infection (Ruggieri et al., 2012).

Finally, in contrast to bacterial infections, a cell line infected with a protozoan parasite *Plasmodium* did not result in host SG formation at 1 hpi as assessed by multiple SG marker proteins, including G3BP1, eIF4G, and eIF3η (Hanson and Mair, 2014). Furthermore, extending the infection time up to 24 h, did not reveal SG formation.

Taken together, these results reveal that host SG assembly, when examined, can be a response to microbial infection. In addition, from the handful of microbes investigated, there appears to be mechanisms to limit the extent of SG formation. Nevertheless, a limitation of these studies is that mostly a single SG marker protein (TIA1) was used, precluding identification of compositional differences upon infection. For example, SG compositional differences have been often observed in viruses, where non-SG foci can assemble with the SG protein G3BP1 (Eiermann et al., 2020). However, since host eIF2α phosphorylation is a common outcome of microbial infection, it should be expected that SG assembly is a similarly widespread host response as described below.

## eIf2α Phosphorylation and Microbial Infection

The proximal cause of canonical physiological SG assembly is generally phosphorylation of the α subunit of eIF2 (eIF2α). eIF2 is a translation initiation factor, that, when bound to GTP brings the initial Met-tRNA_*i*_^*Met*^ to the ribosomal pre-initiation complex beginning cap-dependent translation in eukaryotes (Adomavicius et al., 2019). After initial AUG recognition, eIF2-GDP is generated through hydrolysis, activated by the GTPase-activating protein eIF5. To facilitate further translation initiation, eIF2-GTP must be re-generated by interaction with its guanine nucleotide exchange factor, eIF2B. Phosphorylation of eIF2α increases its affinity to eIF2B, which both prevents the GDP:GTP exchange and sequesters eIF2α in a high-affinity complex (Hershey, 1989). An elevation of P-eIF2α by 20–30% has been suggested to be sufficient for sequestration of eIF2B to limit translation initiation, depending on cell type and organism (Brostrom and Brostrom, 1997). These combined effects inhibit translation initiation and promote SG formation.

This review will concentrate on physiologically predominant SGs that are reliant on P-eIF2α assembly, termed “type I” (Riggs et al., 2020; Hofmann et al., 2021). However, other modes of assembly have been documented with some differences in protein composition: for example, type II SG assembly occurs due to inhibition of eIF4A activity, while type III SGs are induced by nitric oxide, UV, as well as other agents (Hofmann et al., 2021). In addition, there remains an additional class of SGs induced by molecular crowding, for example, by osmotic stress (Bounedjah et al., 2012; Jalihal et al., 2020). These additional cases are significantly less studied compared with the canonical type I SG that is the focus of this review this review.

**FIGURE 1:**
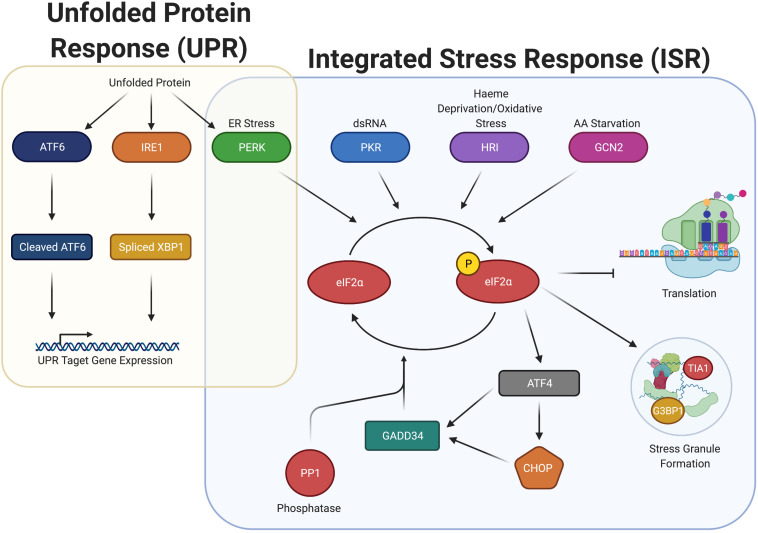
Integrated stress response (ISR) increases P-eIF2α and induces SGs, but overlaps with the unfolded protein response (UPR) through the activation of PERK. ISR detects ER Stress, dsRNA, heme deprivation and oxidation stress, and amino acid starvation and promote the dimerization and activation of the stress kinases PERK, PKR, HRI, and GCN2, respectively. Once activated, these kinases phosphorylate eIF2α, which stalls translation and promotes the formation of stress granules. P-eIF2α also promotes the selective translation of uORF containing mRNA, including ATF4, CHOP, and GADD34. GADD34 associates with the eIF2α phosphatase PP1 to dephosphorylate eIF2α during stress recovery. The ISR is closely associated with the UPR, connected through PERK. Upon detection of unfolded proteins, ATF6, IRE1, and PERK are all activated, promoting cleavage of ATF6, splicing of XBP1, and the induction of P-eIF2α. Cleaved ATF6 and spliced XBP1 promote the expression of UPR target genes to elicit a response to unfolded protein.

Phosphorylation of eIF2α occurs in response to diverse stresses, which cause the pathway activation to be termed the integrated stress response (ISR). The ISR is regulated by four eIF2α kinases in humans: PKR, PERK, GCN2, and HRI (discussed in their own section below). Activation of these eIF2α kinases induce phosphorylation of eIF2α at serine 51 (Figure 1).

Assembly of *bona fide* SGs due to infection, results from eIF2α phosphorylation (P-eIF2α) with few exceptions as described previously. A useful proxy for the presence of SGs due to infection can be elevated P-eIF2α or the activation of the kinases that phosphorylate eIF2α. In the absence of data demonstrating SGs during bacterial, fungal, and protozoan infection, the activation of ISR kinases, and/or elevated P-eIF2α can provide a suggestive view into whether SGs form.

**FIGURE 2:**
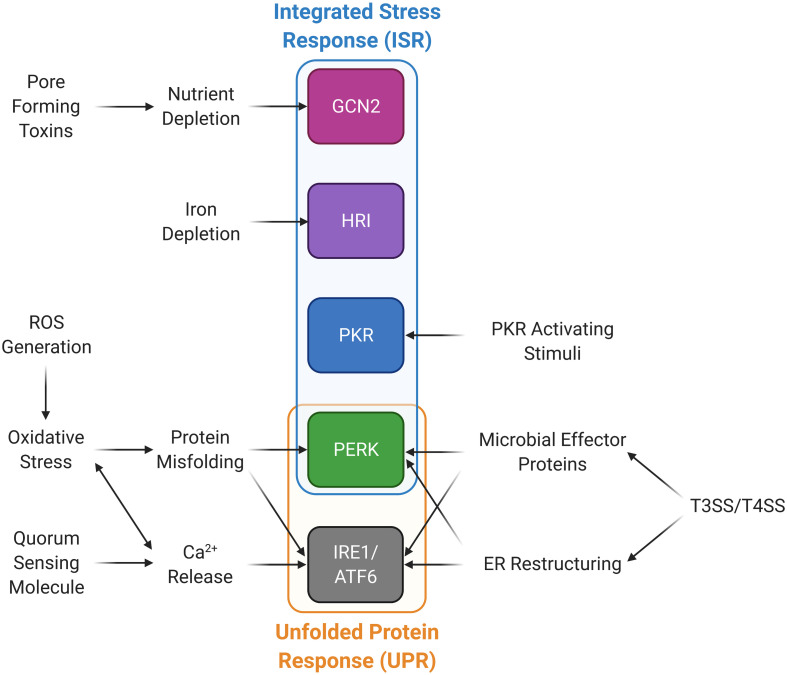
Microbial stimuli activate stress kinases in the integrated stress response (ISR) and the unfolded protein response (UPR) pathways. Microorganisms are able to induce the ISR and UPR through a variety of mechanisms. Type III and IV bacterial secretion systems (T3SS and T4SS) promote ER restructuring and the release of microbial effector proteins that can activate PERK and the UPR. PKR can be activated through PKR activating stimuli other than dsRNA. Calcium can be released through quorum sensing molecules and ROS generation, which also induces oxidative stress and protein misfolding, in turn activating the UPR and PERK. Nutrient and iron deprivation may be induced by microbes and their products promoting activation of GCN2 and HRI, respectively.

Many studies examining P-eIF2α levels have identified individual bacterial and fungal proteins, and small molecules that are responsible for the increase in the proportion of P-eIF2α (Table 2). These molecules also give insight into the mechanism by which microbes are sensed in the host cell. Most prominent among these are the formation of pores in the host membrane, host membrane damage, and reactive oxygen species (ROS). There are also other mechanisms and cases where no mechanism is known, simply that the infection by the microbe itself results in an increase in host P-eIF2α (Figure 2 and Table 3).

**TABLE 2:** Microbial molecules that increase eIF2α phosphorylation.

Mechanisms	Microbe (Compound)	Type	References
**Pore forming protein**	*Aeromonas hydrophila (PA)*	*Gram (−)*	Gonzalez et al. (2011)
	*E. coli (hemolysin A)*	*Gram (−)*	Kloft et al. (2010)
	*Vibrio cholerae (VCC cytolysin)*	*Gram (−)*	Kloft et al. (2010)
	*Listeria monocytogenes (Listeriolysin O)*	*Gram (+)*	Gonzalez et al. (2011); Pillich et al. (2012); Tattoli et al. (2013)
	*Staphylococcus aureus* (α*-toxin)*	*Gram (+)*	Kloft et al. (2010); von Hoven et al. (2012)
	*Streptococcus pyogenes (Streptolysin O)*	*Gram (+)*	Kloft et al. (2010)
**Membrane damage**	*Salmonella typhimurium (Type III secretion system)*	*Gram (−)*	Kloft et al. (2010); Vonaesch et al. (2016)
	*Shigella flexneri (Unknown)*	*Gram (−)*	Tattoli et al. (2012)
	*Mycobacterium tuberculosis (ESAT-6, membrane lysis)*	*Mycobacteria*	Choi et al. (2010)
**ROS/ROS generation**	*Mycobacterium tuberculosis (Heparin-Binding Hemagglutinin Adhesin-HBHA)*	*Mycobacteria*	Choi et al. (2013)
	*Mycobacterium tuberculosis (38-kDa antigen)*	*Mycobacteria*	Lim et al. (2015)
	*Pseudomonas aeruginosa (Pyocyanin)*	*Gram (−)*	Yang et al. (2016)
	*Streptococcus pneumoniae (Pneumococcal H_2_O_2_)*	*Gram (+)*	Loose et al. (2015)
**ER stress through BiP cleavage**	*Shiga toxigenic E. coli (Subtilase cytotoxin)*	*Gram (−)*	Morinaga et al. (2008); Wolfson et al. (2008)
**Other mechanisms**	*Pseudomonas aeruginosa (Homoserine lactose-HSL-C12)*	*Gram (−)*	von Hoven et al. (2012); Grabiner et al. (2014)
	*Chlamydia trachomatis* (pORF5)	*Gram (−)*	Wen et al. (2020)
	*Histoplasma capsulatum (Calcium binding protein Cbp1)*	*Fungi*	English et al. (2017)

**TABLE 3:** Microbial infections or compounds that affect eIF2α phosphorylation.

Microbe	Induction time	Fold induction	References
**Gram-negative bacteria**			
*Aeromonas hydrophila*	2–7 h pore forming toxin exposure	3x	Gonzalez et al. (2011)
*Campylobacter jejuni*	9–12 hpi	3x	Tentaku et al. (2018)
*Chlamydia pneumoniae*	2 hpi (persistent infection only)	2x	Shima et al. (2015)
*Chlamydia trachomatis*	12 hpi	2x	Wen et al. (2020)
*Chlamydia trachomatis*	24–45 hpi	+	Ohmer et al. (2019)
*E. coli - Shiga toxigenic (STEC)*	1 h subtilase exposure	+++	Morinaga et al. (2008); Wolfson et al. (2008)
*Pseudomonas aeruginosa*	1–2 h pyocyanin exposure	2x	van ‘t Wout et al. (2015)
*Shigella flexneri*	2 hpi	+	Vonaesch et al. (2016)
	1 hpi	+++	Tattoli et al. (2012)
*Vibrio vulnificus*	3–6 h pore forming toxin exposure	+++	Song et al. (2016)
*Yersinia pseudotuberculosis*	1 hpi	+++	Shrestha et al. (2012)
**Gram-positive bacteria**			
*Clostridium difficile*	42 hpi of mice	2x	Sadighi Akha et al. (2013)
	12–18 h toxin B exposure	2x	Sun et al. (2014)
*Listeria monocytogenes*	0.5–2 hpi	+++	Gonzalez et al. (2011); Shrestha et al. (2012); Tattoli et al. (2012)
	10 hpi	++	Besic et al. (2020)
	4–6 hpi	+	Pillich et al. (2012)
*Staphylococcus aureus*	80–120 min α-toxin exposure	+++	Kloft et al. (2010)
*Streptococcus pneumoniae*	3–5 hpi	+++	Loose et al. (2015)
**Mycobacteria**			
*Mycobacterium avium*	12–24 hpi (>5 MOI)	10x	Go et al. (2019)
*Mycobacterium tuberculosis*	8 weeks post infection (granulomas)	Immunofluor.	Seimon et al. (2010)
	6–24 h 38-kDa antigen exposure	+++	Lim et al. (2015)
	12–24 h hemagglutinin antigen exposure	+++	Choi et al. (2013)
*Mycobacterium ulcerans*	12–24 h 38-kDa antigen exposure	+++	Ogbechi et al. (2018)
*Mycobacterium tuberculosis*	6–24 h Ag 38 kDa antigen exposure	10x	Lim et al. (2015)
**Fungi**			
*Histoplasma capsulatum*	12 hpi (5 MOI)	2x	English et al. (2017)
**Protozoa**			
*Leishmania amazonensis*	2–4 hpi	+++	Pereira et al. (2010)
*Plasmodium berghei*	7 days post infection in mouse brains	3x	Anand and Babu (2013)
*Toxoplasma gondii*	3 hpi	++	Ietta et al. (2017)

**Reduction in eIF2α phosphorylation**			

**Microbe**	**Induction time**	**Fold *reduction***	**References**

**Gram-negative bacteria**			
*Simkania negevensis*	3 days post infection	−10x	Mehlitz et al. (2014)
**Other bacteria**			
*Mycobacterium tuberculosis*	6–24 hpi	-/–	Lim et al. (2011)
*Mycoplasma hyopneumoniae*	6–24 hpi	-	Pan et al. (2020)

*+,++, +++ are subjective indications of increased P-eIF2α due to absence of quantification in study (∼2x, ∼4x, ∼10x), -, – are subjective indications of decreased P-eIF2α. PFT, pore-forming toxin.*

It is striking that infection results in elevated P-eIF2α in most cases examined. It is important to note that in many cases, the direct effect on P-eIF2α has not been measured, but only inferred from downstream effects on translation. While P-eIF2α inhibits general translation initiation, it preferentially increases translation of mRNAs containing upstream open reading frames in their 5’ UTR by multiple mechanisms (Wek, 2018). Many of these preferentially translated proteins have central roles in the stress response signaled by P-eIF2α, such as GADD34, ATF4, and CHOP, which among other proteins, are typically used as readouts for the ISR (see Figure 1). Their roles in stress are diverse. ATF4 is a transcription factor promoting transcription of genes in the ISR including GADD34 and CHOP. GADD34 is a scaffolding protein that promotes dephosphorylation of P-eIF2α by targeting the PP1 phosphatase to P-eIF2α, whereas CHOP is a pro-apoptotic transcription factor whose expression is also dependent on ATF6, another ER stress-induced transcription factor.

### Gram-Negative Bacteria

Most studies on host P-eIF2α levels during infection have been performed on Proteobacteria, a diverse Gram-negative phylum containing a number of human pathogens. Among these bacteria, all studies described here have shown increased host P-eIF2α in response to infection or metabolites produced by the bacteria in human cells.

Gammaproteobacteria is the largest class of Proteobacteria and includes *Escherichia coli*, the most well-studied bacteria of this grouping. The Shiga toxin-producing strain (STEC), capable of causing gastroenteritis in humans, promotes an increase in P-eIF2α via expression of subtilase cytotoxin (Morinaga et al., 2008; Wolfson et al., 2008). These studies demonstrated increased P-eIF2α in Vero cells that occurs in response to addition of the toxin. Subtilase is a protease that when internalized, mediates cleavage of the ER chaperone BiP, which is important in monitoring ER stress and results in activation of eIF2α kinase PERK and induction of CHOP (Figure 1). However, the effect was only seen when subtilase was added to the cells and not that of the infection with the organism itself.

Other gammaproteobacteria elevate host P-eIF2α through pore-forming toxins (PFTs). For example, exposure to aerolysin PFT from *Aeromonas hydrophila* in HT29 cells promotes a ∼threefold increase in P-eIF2α after 3.5 h (Gonzalez et al., 2011). This is comparable with the VvhA toxin in the gammaproteobacteria *Vibrio vulnificus*, which induces P-eIF2α in Caco-2 cells to nearly threefold after 6 h of exposure (Song et al., 2016). In addition, similar to the STEC bacteria, the expression of CHOP and ATF4 was also increased.

Primary cells were also shown to activate the ISR when exposed to microbial toxins. Primary bronchial epithelial cells incubated with cultured media from the Gammaproteobacteria *Pseudomonas aeruginosa* PAO1 strain resulted in a twofold increase in P-eIF2α after 4 h of exposure (van ‘t Wout et al., 2015). Further investigation identified two toxins as sufficient for the increase: the alkaline protease AprA that can affect protein translocation in the ER and pyocyanin, which generates ROS in host cells.

A number of other gammaproteobacteria show similar upregulation of P-eIF2α in host cells upon infection. Elevated P-eIF2α was reported for *Yersinia pseudotuberculosis* infection of the mouse macrophage-like RAW 264.7 cells (Shrestha et al., 2012). This study further investigated downstream gene expression and determined that an ATF4 reporter was induced in HEK293 cells infected with *Y. pseudotuberculosis*, along with a similar expression in RAW 264.7 cells (Shrestha et al., 2012).

*Shigella flexneri* illustrates a clear role of infection in elevated host P-eIF2α. HeLa cells exhibit increased P-eIF2α levels at 1–2 hpi (Tattoli et al., 2012; Vonaesch et al., 2016; Abdel-Nour et al., 2019). Nevertheless, within 4 hpi, P-eIF2α returns to near uninfected levels (Tattoli et al., 2012). These data suggest that *S. flexneri* may possess a mechanism to temper this response, possibly via the GADD34 protein that regulates PP1 phosphatase to target P-eIF2α.

While P-eIF2α levels were not investigated for the closely related bacteria, *Salmonella typhimurium*, they are likely to be elevated in host cells during its infection as well. Evidence for elevated P-eIF2α includes activation of an upstream P-eIF2α kinase, along with downstream gene induction, and even the formation of SGs in HeLa cells upon infection, which largely occurs via the P-eIF2α pathway (Tattoli et al., 2012).

Another example in Proteobacteria is *Campylobacter jejuni*, an epsilonproteobacteria, which has a near threefold increase in P-eIF2α after 12 h of infection in Caco-2 cells when compared with uninfected cells (Tentaku et al., 2018).

Finally, elevated P-eIF2α was observed in the Gram-negative bacteria *Chlamydia pneumonia* and *Chlamydia trachomatis* infection. As an obligate intracellular bacteria, the infection of a host cell and bacterial replication occur differently in *Chlamydia* compared with the other bacteria described here (Moulder, 1991; Hanada et al., 2003; Mpiga and Ravaoarinoro, 2006). They exhibit a biphasic developmental cycle, in which they produce small elementary bodies (EBs) which are extracellular, infectious, metabolically inactive, and non-dividing. Once infecting a cell, EBs can differentiate into much larger intracellular reticulate bodies (RBs) that can divide and multiply within membrane-limited chlamydial inclusions. The replicating RBs can be transformed into EBs to be distributed upon host cell lysis.

Chlamydial productive infection results in lysis within 48–72 hpi. However, during bacterial stress and in certain serovars, a persistent form of infection can occur that has low infectivity, retaining the RB form of *Chlamydia* (Moulder, 1991; Hanada et al., 2003; Mpiga and Ravaoarinoro, 2006). These infections show a similar response with P-eIF2α in host cells as to the other bacteria described above. *C. pneumonia* was shown to induce P-eIF2α nearly twofold in HEp-2 cells after 2 h of persistent infection (Shima et al., 2015), while *C. trachomatis* promoted P-eIF2α to a similar fold increase at 12, 24, and 40 h post persistent infection in HeLa cells along with the corresponding upregulation of the downstream genes, ATF4 and CHOP (Wen et al., 2020). In contrast, a lymphogranuloma venereum serovar that causes a productive, invasive infection did not cause elevated P-eIF2α at 2.5 hpi (Böhme et al., 2010).

*Simkania negevensis* is an obligate intracellular bacteria, closely related to *C. trachomatis* and *C. pneumonia*, that causes no significant changes in P-eIF2α in response to its infection at 48 hpi (Mehlitz et al., 2014). However, as noted above, P-eIF2α elevation may be transient during infection, and thus potentially only noticeable earlier in infection. Interestingly, *S. negevensis* also inhibited P-eIF2α production from drug-induced ER stress by thapsigargin; therefore, it is possible that this bacteria may be capable of directly preventing increased P-eIF2α levels early in infection.

### Gram-Positive Bacteria

Infection of Gram-positive bacteria similarly elevates P-eIF2α as seen in Gram-negative bacteria; however, these species have been examined to a more limited extent. The species examined for this effect are all members of the Firmicutes phylum, either Bacilli (*Listeria monocytogenes*, *Streptococcus pneumoniae*, and *Staphylococcus aureus*) or Clostridia (*Clostridium difficile*).

*Listeria monocytogenes*, a pathogenic rod-shaped bacterium, is one of the most well-studied Gram-positive bacterium with regard to P-eIF2α. *L. monocytogenes* can actively penetrate host cell membranes and is the most common cause of listeriosis, a disease that can lead to severe illness and even death. Elevated P-eIF2α in cultured cells has been observed with *L. monocytogenes* from both infection and exposure to *Listeria*-specific toxins. In mouse cells, infection with *L. monocytogenes* resulted in both elevated P-eIF2α and expression of the downstream P-eIF2α-regulated genes (Shrestha et al., 2012). An investigation of *L. monocytogenes* infection also demonstrated that P-eIF2α increases after bacterial infection in the P388D1 mouse macrophage cell line (Pillich et al., 2012). Similarly, an increase in P-eIF2α was observed in HeLa cells infected with *L. monocytogenes* between 0.5 and 2 h, with a decrease to near basal uninfected levels after 3 h (Tattoli et al., 2013).

Listeriolysin O (LLO), a pore-forming toxin (PFT) produced by *L. monocytogenes*, is sufficient for the increase in P-eIF2α levels in P388D1 and HT29 cells after 1 and 2 h, respectively (Gonzalez et al., 2011; Pillich et al., 2012). Supporting these results, was the absence of a significant increase in P-eIF2α when infected with a *L. monocytogenes* strain lacking the gene encoding for LLO (Pillich et al., 2012; Besic et al., 2020).

Another PFT was the causative agent for increased P-eIF2α in the bacillus *Staphylococcus aureus* (Kloft et al., 2010). *S. aureus* is commonly found in skin flora and associated with a number of human diseases. It was shown to elevate P-eIF2α levels by exposure to the pore-forming α-toxin in as little as 40 min in HaCaT cells (Kloft et al., 2010).

In contrast with the other Firmicutes, a PFT from the bacillus *Streptococcus pneumoniae* does not elevate P-eIF2α (Table 3). The amount of P-eIF2α increased in H441 cells upon incubation with *S. pneumoniae* supernatant and at 3 hpi (Loose et al., 2015). This effect was abrogated by addition of catalase to the supernatant as well as in an infection with a bacterial mutant deficient in hydrogen peroxide production, suggestive of being caused by streptococcal hydrogen peroxide.

Finally, *Clostridium difficile*, which is a more distantly related Gram-positive rod-shaped Firmicutes that causes antibiotic-related gastrointestinal illnesses, was shown to induce elevated P-eIF2α in infected mouse cells, along with increased downstream GADD34 expression (Sadighi Akha et al., 2013). Furthermore, it was found that the secreted *Clostridium* toxin B, which also disrupts host intracellular signaling and cell structure, induced P-eIF2α in mouse CT26 cells, along with increased expression of the downstream genes ATF4 and CHOP as well as ATF6, which belongs to the UPR (Sun et al., 2014).

### Mycobacteria and Other Bacteria

*Mycobacterium*, which falls within the Gram-positive phylum Actinobacteria, is characterized by “acid fastness” which prevents Gram-positive staining due to the lipids attached to the cell wall. However, *Mycobacterium* appears to be no different in their ability to induce P-eIF2α in host cells.

*Mycobacterium avium*, a human pathogen that can cause severe illness in immunocompromised patients (Kiehn et al., 1985), showed an increase in P-eIF2α between 12 and 24 hpi in RAW 264.7 cells (Go et al., 2019). They also showed an induction of the upstream activating kinase PERK prior to the increase in eIF2α phosphorylation, along with increased downstream CHOP expression coinciding with elevated P-eIF2α.

*Mycobacterium ulcerans*, capable of causing Buruli ulcer, produces the toxin mycolactone, which was shown to induce P-eIF2α when exposed to HeLa cells, along with increased CHOP and ATF4 protein expression (Ogbechi et al., 2018).

*Mycobacterium tuberculosis*, likely the most infamous *Mycobacterium* due to being the causative agent of tuberculosis, is one organism that appears to both increase and decrease P-eIF2α (Table 3). One study showed that RAW 264.7 cells infected with *M. tuberculosis* inhibited P-eIF2α at 24 hpi, but also observed significant expression of the P-eIF2α downstream gene CHOP at this time point (Lim et al., 2011). The study further found that two *M. tuberculosis* virulence factors, the 38-kDa antigen and the heparin-binding hemagglutinin antigen (HBHA), were capable of inducing P-eIF2α and downstream CHOP protein expression in both BMDM and RAW 264.7 cells (Choi et al., 2013; Lim et al., 2015). This is interesting, as it would suggest that certain molecules expressed by *M. tuberculosis* promote elevated P-eIF2α, while others counteract this response. Finally, in granulomas of *M. tuberculosis*-infected mice, increased P-eIF2α was detected along with increased gene expression in P-eIF2α downstream genes (Seimon et al., 2010).

Taken together, these results demonstrate that Mycobacteria may have an adaptation to reduce elevated P-eIF2α after infection. While reduction of P-eIF2α in *M. tuberculosis*-infected cells commenced at 24–36 h, it was not reported for *M. avium*. However, it is possible that extended infection could also result in P-eIF2α reduction in this case as well.

Finally, the unrelated bacteria *Mycoplasma hyopneumoniae*, which has the unusual characteristic of lacking a cell wall, has shown a similar effect on P-eIF2α (Pan et al., 2020). This study showed a decrease in the P-eIF2α to 20% of the total eIF2α after 36 h, along with a decrease in ATF4 mRNA levels over the same time frame.

### Eukaryotic Microbial Infections

Elevated P-eIF2α has also been observed in fungal and protozoan infection. However, very few eukaryotic organisms have been investigated for elevated P-eIF2α upon infection, limited to three protozoan species (*Leishmania amazonensis*, *Plasmodium berghei*, and *Toxoplasma gondii*) and one fungal species (*Histoplasma capsulatum*).

The three protozoan species all demonstrated an increase in P-eIF2α upon infection of both mice and cultured cells. First, *Leishmania amazonensis*, a trypanosome that causes leishmaniasis, was shown to induce P-eIF2α in THP-1 cells, along with PKR phosphorylation from 2 to 4 hpi (Pereira et al., 2010). Second, *Plasmodium berghei*, a protozoan parasite, which causes malaria in rodents, was found to cause elevated P-eIF2α in mouse brains 7 days after intraperitoneal infection (Anand and Babu, 2013). Third, *Toxoplasma gondii*, a protozoan parasite that can cause toxoplasmosis in humans, was shown upon infection of BeWo cells to cause a near fourfold increase in P-eIF2α compared with uninfected cells 24–48 hpi (Ietta et al., 2017).

*Histoplasma capsulatum*, which causes the disease histoplasmosis is the lone fungal pathogen examined for P-eIF2α levels. This fungus promotes P-eIF2α increases in infected macrophages 12 hpi to a similar extent to that caused by tunicamycin treatment (English et al., 2017). This study also found elevation of downstream ATF4 protein and CHOP mRNA expression in the first 24 h of lytic infection (English et al., 2017).

### Adaptation to Microbial Infections

The vast majority of microbial infections examined result in elevated host P-eIF2α. However, at least four species are exceptions to this outcome. Since P-eIF2α has not been extensively examined during microbial infection, these outliers may represent common adaptations of either the host or microbe. These examples fall into three categories: return to basal uninfected P-eIF2α levels, no increase in P-eIF2α, or a decrease in P-eIF2α.

First, a transient increase in P-eIF2α with return to basal levels was observed in both Gram-positive and Gram-negative bacteria. For example, in the Gram-positive bacteria *Listeria monocytogenes*, a transient increase in P-eIF2α was observed after 30 min, returning to near basal uninfected levels after 3 h (Tattoli et al., 2013). This also occurred within 3–4 h after *Shigella* infection (Tattoli et al., 2012), 7 h after *Aeromonas* infection (Gonzalez et al., 2011), and after 2–4 h of shiga toxigenic *E. coli* infection (Morinaga et al., 2008; Wolfson et al., 2008).

Second, no increase in P-eIF2α was observed upon infection with the Gram-negative bacteria, *Simkania negevensis*, when examined at 3 hpi (Mehlitz et al., 2014).

Finally, the mycobacterial species *M. tuberculosis* and *Mycoplasma* species *M. hyopneumoniae*, both reduced the level of P-eIF2α at 24–36 hpi (Lim et al., 2011; Pan et al., 2020).

Taken together, these examples may represent unique microbial adaptation strategies to limit elevated host P-eIF2α. However, it is possible that most microbial infections cause a return to uninfected host P-eIF2α levels as the infection progresses and can even lower the P-eIF2α below that of uninfected levels as seen in mycobacteria. More study with longer time-courses will be necessary to resolve this issue in the future.

## Activation of the Integrated Stress Response Upon Microbial Infection

In mammals, multiple cellular stresses are sensed by four eIF2α kinases (Pakos-Zebrucka et al., 2016). Activation of an eIF2α kinase can provide clues to the type of stress that is occurring in the infected cells. The common stresses that are known to induce P-eIF2α during infection are ER stress and amino acid starvation (Celli and Tsolis, 2015; Abdel-Nour et al., 2019). These are primarily sensed by two different eIF2α kinases: PERK (PKR-like endoplasmic reticulum kinase/EIF2AK3) and GCN2 (general control non-derepressible 2/EIF2AK4). The other two eIF2α kinases are: HRI (heme-regulated inhibitor/EIF2AK1) and PKR (double-stranded RNA-activated protein kinase R/EIF2AK2).

Specific cellular stresses will activate these kinases, although there is considerable overlap resulting in activation of multiple kinases (Figure 2). HRI is activated by heme deprivation in erythrocytes as well as arsenite−induced oxidative stress, heat shock, and osmotic stress in other cells (Burwick and Aktas, 2017). PKR is most commonly induced by viral infection as it is activated by double-stranded RNA, which is often present as a result of viral replication (García et al., 2006). A diverse set of other stresses also can activate PKR as well. Activation of PERK by ER stress with its most well-defined mechanism being dissociation of the Hsp70 protein BiP/GRP78 due to unfolded proteins (Kopp et al., 2019). Finally, GCN2, which is the most widely conserved kinase, found from yeast to humans, is activated during amino acid starvation by detection of deacylated tRNA (Masson, 2019). In microbial infection, these kinases can be activated by either intracellular microbial infection, or extracellular microbes, presumably from molecules produced by the microbe in question (Table 4).

**TABLE 4:** Activation of eIF2α kinases by microbes.

Kinase	Microbe	Stress origin	Type	References
**PERK**				
	*Pseudomonas aeruginosa*	Extracellular (C12-HSL)	*Gram (−)*	Grabiner et al. (2014)
	*Listeria monocytogenes*	Extracellular (Listeriolysin O - LLO)	*Gram (+)*	Pillich et al. (2012)
	*Streptococcus pneumoniae*	Extracellular (H_2_O_2_)	*Gram (+)*	Loose et al. (2015)
	*Mycobacterium ulcerans*	Extracellular (Mycolactone)	*Mycobacteria*	Ogbechi et al. (2018)
	*Plasmodium berghei*	Intracellular	*Protozoa*	Anand and Babu (2013)
**PERK (Including UPR/CHOP/IRE1 activation)**				
	*Brucella melitensis*	Intracellular (TcpB)	*Gram (−)*	Smith et al. (2013)
	*Brucella abortus*	Extracellular (VceC via Type IV secretion system)	*Gram (−)*	de Jong et al. (2013)
	*Campylobacter jejuni*	Intracellular	*Gram (−)*	Tentaku et al. (2018)
	*Chlamydia muridarum*	Intracellular (Extra protein expression, flow of ATP and nutrients into the inclusion)	*Gram (−)*	George et al. (2017)
	*Chlamydia trachomatis*	Intracellular	*Gram (−)*	Shima et al. (2015); George et al. (2019); Wen et al. (2020)
	*E. coli - Shiga toxigenic (STEC)*	Extracellular (Subtilase cytotoxin)	*Gram (−)*	Morinaga et al. (2008); Wolfson et al. (2008)
	*Shigella dysenteriae*	Extracellular (Shiga toxin Stx1)	*Gram (−)*	Lee et al. (2008)
	*Clostridium difficile*	Extracellular (Toxin B)	*Gram (+)*	Sun et al. (2014)
	*Listeria monocytogenes*	Extracellular (Listeriolysin O - LLO)	*Gram (+)*	Pillich et al. (2012); Shrestha et al. (2012)
	*Mycobacterium avium*	Intracellular	*Mycobacteria*	Go et al. (2019)
	*Mycobacterium tuberculosis*	Extracellular (38-kDa antigen)	*Mycobacteria*	Lim et al. (2015)
	*Mycobacterium tuberculosis*	Intracellular	*Mycobacteria*	Lim et al. (2011)
	*Leishmania amazonensi*	Intracellular	*Protozoa*	Dias-Teixeira et al. (2017)
**GCN2**				
	*E. coli*	Extracellular (Hemolysin A)	*Gram (−)*	Kloft et al. (2010)
	*Pseudomonas aeruginosa*	Extracellular (Pyocyanin)	*Gram (−)*	Yang et al. (2016)
	*Salmonella typhimurium*	Intracellular (SPI-2-independent)	*Gram (−)*	Tattoli et al. (2012, 2013)
	*Shigella flexneri*	Intracellular	*Gram (−)*	Tattoli et al. (2012)
	*Vibrio cholerae*	Extracellular (VCC cytolysin)	*Gram (−)*	Kloft et al. (2010)
	*Staphylococcus aureus*	Extracellular (α-toxin)	*Gram (+)*	Kloft et al. (2010)
	*Streptococcus pyogenes*	Extracellular (Streptolysin O - SLO)	*Gram (+)*	Kloft et al. (2010)
	*Listeria monocytogenes*	Intracellular (Listeriolysin O - LLO)	*Gram (+)*	Tattoli et al. (2013)
	*Mycobacterium ulcerans*	Extracellular (Mycolactone)	*Mycobacteria*	Ogbechi et al. (2018)
**PKR**				
	*Chlamydia trachomatis*	Intracellular	*Gram (−)*	Webster et al. (2016)
	*Staphylococcus aureus*	Extracellular (α-toxin)	*Gram (+)*	Kloft et al. (2010)
	*Mycobacterium ulcerans*	Extracellular (Mycolactone)	*Mycobacteria*	Ogbechi et al. (2018)
	*Leishmania amazonensi*	Intracellular	*Protozoa*	Pereira et al. (2010)
	*Toxoplasma gondii*	Intracellular	*Protozoa*	Portillo et al. (2017)
**HRI**				
	*Shigella flexneri*	Intracellular	*Gram (−)*	Abdel-Nour et al. (2019)
	*Pseudomonas aeruginosa*	Extracellular (Pyocyanin, ApaA)	*Gram (−)*	van ‘t Wout et al. (2015)

*Extracellular may refer to microbial compound alone or extracellular microbes.*

An additional stress on the host cell is from the bacterial type III and type IV secretion systems (T3SS, T4SS). These are two examples of mechanisms employed by bacteria to secrete proteins and DNA into host cells aiding infection. T3SS is found in a number of Gram-negative bacteria, including, *S. typhimurium* (Coburn et al., 2007), while T4SS is found in both Gram-negative and Gram-positive bacteria (Grohmann et al., 2018). Each system employs a similar, yet distinct method to permeabilize the host cell membrane that may lead to the induction of a stress response. These systems can also lead to activation of the eIF2α kinases PERK, GCN2, and HRI as described below.

### PERK and the Unfolded Protein Response

Endoplasmic reticulum stress during infection can manifest in several manners, but is generally activated by unfolded proteins and insufficient protein-folding capacity in ER, which induces the unfolded protein response (UPR). In microbial infection, the source of unfolded proteins or ER stress itself is often linked to organisms that can live intracellularly, in close association with ER (Celli and Tsolis, 2015).

The UPR is a three-pronged system (Figure 1), composed of ATF6, IRE1, and PERK (Walter and Ron, 2011). When the UPR is activated, the active forms of ATF6 and IRE1 assist the cell in combating ER stress. In this situation, ATF6 is cleaved and promotes transcription of genes to enhance protein folding capacity, while IRE1 is a kinase that engages in non-conventional splicing of the mRNA encoding for the XBP1 transcription factor. When spliced, XBP1 mRNA is translated into its active form, that drives transcription of genes facilitating ER expansion. Finally, PERK, as described above, phosphorylates eIF2α. Increased P-eIF2α inhibits translation initiation, thus reducing the load on the ER.

In addition to general inhibition of initiation, P-eIF2α promotes preferential translation of ATF4. ATF4, in turn, leads to transcription of CHOP mRNA, that itself promotes both transcription of apoptosis-related genes and the phosphatase-interacting protein GADD34 (Figure 1). GADD34 complexes with the catalytic subunit of the PP1 phosphatase (PP1c) to promote its activity, reducing P-eIF2α, maintaining a lower level of P-eIF2α as high P-eIF2α levels can promote apoptosis. The reduction in P-eIF2α can thus allow protection of the cell from ER stress, while avoiding apoptosis (Walter and Ron, 2011). A number of microbial pathogens induce PERK activation in host cells, either exclusively or alongside the UPR. Therefore, the consequences of infection can be due to P-eIF2α from PERK activation or dependent on the two other branches of the UPR (ATF6 and IRE1).

During microbial infection, the activation of PERK is caused by a variety of mechanisms (Table 4). The two most prominent of these are calcium release from the ER and oxidative stress. There are also other mechanisms, such as BiP cleavage and those that activate PERK by an unknown means.

First, several microbes activate PERK through calcium release from the ER. Calcium release activates ER stress through disruption of Ca^2+^ homeostasis, as many ER chaperones require calcium to function (Schröder, 2008). A likely explanation for the activation of PERK by the Gram-negative bacteria *Pseudomonas aeruginosa* is through the calcium release triggered by the secretion of its quorum-sensing molecule, *N*-(3-oxododecanoyl)-homoserine lactone (HSL-C12) (Grabiner et al., 2014). HSL-C12 likely acts on PERK, as P-eIF2α levels are greater in cells containing PERK than in PERK^–/–^ cells. Nevertheless, some residual elevated P-eIF2α was observed in PERK^–/–^ cells, suggestive of involvement of other eIF2α kinases. Another study found that GADD34 and CHOP expression were induced in infected cells, but this induction was tapered by the knockdown of HRI (van ‘t Wout et al., 2015). Furthermore, *P. aeruginosa* infection was shown to induce P-eIF2α in host cells via GCN2 (Yang et al., 2016). These reports implicate *P. aeruginosa* in activation of multiple kinases upon infection, resulting in elevated P-eIF2α.

The Gram-negative bacteria *Campylobacter jejuni* is another example of increased P-eIF2α and PERK activation upon infection, presumably by Ca^2+^release (Hu et al., 2005; Tentaku et al., 2018). However, in contrast, this does not appear to activate the other UPR-associated responses, such as splicing of XBP1 and cleavage of ATF6 (Tentaku et al., 2018).

Release of Ca^2+^ was also implicated in stress induced by the shiga toxin Stx1 from *Shigella dysenteriae* in THP-1 cells (Lee et al., 2008). Stx1 was shown to induce the ER stress response through all three branches of the UPR: PERK, IRE1, and ATF6 (Lee et al., 2008).

The mechanism of UPR activation by the Gram-positive *L. monocytogenes* is additionally likely due to dysregulation of Ca^2+^ levels, which induces ER stress-specific apoptosis in host cells along with P-eIF2α (Pillich et al., 2012). Although not directly investigated, these results suggest PERK activation via UPR leads to increased P-eIF2α through the production of listeriolysin O (LLO). LLO modulates cellular Ca^2+^ levels, which might lead to dysregulation of ER Ca^2+^ homeostasis (Gekara et al., 2008). Similarly, sublytic levels of LLO can create pores in the plasma membrane, which also results in Ca^2+^ influx (Repp et al., 2002).

Second, oxidative stress can induce protein misfolding and thus activate the UPR and ER stress/PERK (Zhang and Kaufman, 2008). The Gram-positive bacteria, *Streptococcus pneumonia*, induced activation of PERK and P-eIF2α via ER stress caused by pneumococcal H_2_O_2_ (Loose et al., 2015). Since ATF6 and IRE1 activation were not observed, it is likely that the UPR was not activated; rather, it was the ISR alone.

Oxidative stress could be the source of ER stress induced by toxin B, an exotoxin produced by *Clostridium difficile.* The toxin was shown to cause ER stress, detected through the activation of IRE1 and BiP, along with induction of PERK, ATF4, and CHOP mRNA (Sun et al., 2014). The mechanism by which the toxin induces ER stress remains unclear, however, more recent work links it to ROS generation (Frädrich et al., 2016).

ER stress via ROS generation may also occur in *Vibrio vulnificus* infection. This bacteria expresses the virulence factor VvhA, which is a pore-forming toxin shown to induce P-eIF2α and CHOP expression. It remains unclear as to which kinase is activated in order to induce P-eIF2α; however, the production of reactive oxygen species by VvhA was found to play a key role (Song et al., 2016).

Generation of ER stress by ROS was also observed in several mycobacterial species. For example, *Mycobacterium avium*, increases activation of IRE1, ATF6, and PERK following infection of host cells (Go et al., 2019). This study suggested that ER stress was caused by *M. avium* induced ROS, which is also likely to play a role in the suppression of the *M. avium* infection.

Similarly, *Mycobacterium tuberculosis* induced ER stress in host cells detected through the upregulation of BiP, CHOP, P-eIF2α, and spliced XBP1 (Lim et al., 2011, 2015). The former study suggested that live *M. tuberculosis* could cause ER stress by ROS and nitric oxide production in RAW264.7 cells. While the later study suggested that the 38-kDa antigen produced by *M. tuberculosis* specifically increased ER stress and downstream signaling through apoptosis-induced activation of Toll-like receptor 2/4. In this study, it was undetermined as to which kinase was activated in response to *M. tuberculosis* infection; however, as with a similar organism, it is likely that this ER stress at least induces PERK activation, if not additional kinases.

In addition, *Mycobacterium ulcerans* produces the virulence factor mycolactone, which was shown to induce P-eIF2α through the activation of PERK, GCN2, and PKR (Ogbechi et al., 2018). Mycolactone produces ROS in primary keratinocytes, which is suggestive of it inducing PERK as seen for the other mycobacteria (Grönberg et al., 2010). This microbial exotoxin, however, does not induce all branches of the UPR, since IRE1 and ATF6 were not activated (Ogbechi et al., 2018).

Third, BiP cleavage can be a source of PERK/UPR activation in microbial infection. This is exemplified by the shiga toxin-producing *E. coli* (STEC) that expresses the subtilase cytotoxin. Subtilase promotes ER stress detected through the cleavage of BiP (Morinaga et al., 2008). Similarly, addition of subtilase to Vero cells promoted the phosphorylation of PERK and eIF2α, stalling protein synthesis, albeit transiently (Wolfson et al., 2008). They also found that this stress induced the other arms of the UPR (IRE1 and ATF6).

Other mechanisms are demonstrated by the obligate intracellular pathogen *Chlamydia muridarum*, which was shown to induce phosphorylation of PERK and the activation of the other two UPR branches in host cells (IRE1 and ATF6) at 48 hpi (George et al., 2017). This UPR response was thought to be caused by the expression of additional proteins in the ER (i.e., *Chlamydia* early genes), flow of ATP and nutrients into the chlamydial inclusion or by binding to BiP.

Furthermore, *Chlamydia*, specifically *C. trachomatis* and *C. pneumonia*, have been shown to activate the UPR through microbial effector protein interaction with the ER (Shima et al., 2015; George et al., 2019). It was shown that *Chlamydia* induces this response through the type III secretion system (T3SS), which facilitates transfer of a number of effector proteins that modify the host cytoskeleton (George et al., 2019). Following this, the *C. trachomatis* protein pORF5 activated PERK, along with UPR-associated proteins, and P-eIF2α, suggesting that both the UPR and ISR are activated upon *Chlamydia* infection (Wen et al., 2020). Furthermore, it was also determined that ER stress induced by *Chlamydia* infection promoted PKR activation via the Toll-like receptor signaling, possible due to IRE1-dependent host degradation activating PKR alongside PERK (Webster et al., 2016).

An alternative mechanism that induces the UPR is found upon infection with the Gram-negative facultative intracellular pathogen *Brucella melitensis*. The bacteria fuses with the ER upon infection, in order to facilitate replication; however, this promotes ER restructuring and induces the ER stress response (Smith et al., 2013). Similarly, infection with *B. melitensis* activated ISR-independent branches of the UPR as shown by upregulated expression of BiP, CHOP, and increased splicing of XBP1. The increased expression of CHOP suggests that P-eIF2α is also induced; however, it remains unclear as to which kinase is activated upon infection, although as with the previous microbial infections, it is likely that the UPR and ER stress are tightly associated with PERK activation.

*Brucella abortus*, which expresses the protein VceC, a type IV secretion system (T4SS) substrate, was shown to induce XBP1 splicing, and interact directly with BiP (de Jong et al., 2013). This would suggest that the UPR is activated upon infection, promoted by ER stress, along with activation of PERK and elevated P-eIF2α, however, this remains to be determined.

Finally, activation of PERK was found after infection with the protozoan parasite *Plasmodium berghei.* This organism was shown to induce PERK activation, along with IRE1 phosphorylation and ATF6 cleavage, in a mouse model (Anand and Babu, 2013). Additionally, eIF2α was also activated upon *P. berghei* infection along with induction of ATF4 and GADD34. Taken together, these results suggest that both the UPR and ISR were activated through ER stress.

### Protein Kinase R

PKR activation is commonly caused by viral infection, as dsRNA triggers PKR trans-autophosphorylation forming an active dimer, which can in turn phosphorylate eIF2α. PKR may also be activated by its activating protein, PACT (protein activator of the interferon-induced protein kinase). However, PKR activation by non-viral microbes, would suggest a novel mechanism for induction of P-eIF2α and stalling of protein synthesis. In contrast to PERK, there are relatively fewer examples of modulation of PKR activation during microbial infection, predominantly in protozoan infections and with the Gram-negative bacteria *Yersinia* (Table 4).

Protein kinase R activation was observed in eukaryotic protozoan parasite infections such as *Leishmania amazonensis*. This parasite activates both host PKR and PERK pathways, resulting in elevated P-eIF2α (Pereira et al., 2010; Dias-Teixeira et al., 2017). Interestingly, in response to infection, the first study speculates that the activation of PKR may be due to increased expression of PKR activating stimuli other than dsRNA (Pereira et al., 2010), while the later study found that XBP1 splicing also occurred following infection with *L. amazonensis* suggesting that the UPR pathway was activated along with PKR/PERK/P-eIF2α activation (Dias-Teixeira et al., 2017).

The protozoan parasite, *Toxoplasma gondii*, appears to also activate PKR and P-eIF2α (Portillo et al., 2017). However, this only occurred with the knockdown of the focal adhesion kinase (FAK), suggesting that the organism induces a mechanism to evade the ISR and host autophagy (Portillo et al., 2017).

In contrast to protozoan activation of PKR, the *Y. pseudotuberculosis* virulence factor YopJ was implicated in the inhibition of PKR, HRI, and PERK activation in MEF cells (Shrestha et al., 2012). Paradoxically, while YopJ inhibits the activation of these kinases, this same study found that *Y. pseudotuberculosis*-infected cells have elevated P-eIF2α. YopJ expression in MEFs exhibited an inhibitory effect on three of the four eIF2α kinases, assessed by reduced ATF4 expression upon PERK, HRI, and PKR activation by kinase-specific-activating drugs in the presence of YopJ. This group had previously found that the *Y. pseudotuberculosis* virulence factor YpkA, increased P-eIF2α in fission yeast cells (Wiley et al., 2009). YpkA is closely associated with YopJ, and both proteins enter host cells via the T3SS. Taken together, these results are suggestive of YopJ functioning to evade the ISR, while alternative virulence factors, likely YpkA, are inducing elevated P-eIF2α; however, it is unclear as to which eIF2α kinase is responsible.

### General Control Non-derepressible 2 and Heme-Regulated Inhibitor

The eIF2α kinases GCN2 and HRI can be activated by multiple stresses. GCN2 is activated by amino acid starvation during infection by a number of organisms that express pore forming toxins, whereas HRI, activated by heme deprivation in erythrocytes, is the least investigated stress kinase in the ISR (Burwick and Aktas, 2017). However, in the few organisms where its activation was observed, it was coupled with the activation of another kinase, suggesting that HRI activation may occur in tandem with other stresses and more commonly activated during infection than previously believed.

Activation of GCN2 has been proposed to be a common host response due to amino acid starvation, first observed in *L. monocytogenes* along with elevated P-eIF2α levels (Tattoli et al., 2012). This is in contrast to the proposal of ER stress and PERK activation previously implicated by elevated P-eIF2α and triggering UPR (ATF6 and IRE1 activation and XBP1 splicing), although PERK activation was not itself examined in this case (Pillich et al., 2012).

Similar mechanisms also appear to occur in pore-forming toxin (PFT)-expressing microbes, which were suggested to activate GCN2 via membrane damage-induced nutrient depletion (Kloft et al., 2010). One such organism, *Staphylococcus aureus*, expresses the PFT α-toxin that was shown to activate GCN2, and interestingly, also PKR; however, the mechanism of activation of this kinase remains unclear (Kloft et al., 2010). They also showed a similar activation of GCN2 in response to other PFTs expressed by microorganisms including *Vibrio cholerae* (cytolysin), *Streptococcus pyogenes* (streptolysin), and *Escherichia coli* (hemolysin A). This would suggest that other organisms producing PFT may share a similar mechanism in promoting GCN2 activation, P-eIF2α, and potentially, SG formation.

Host membrane damage can also cause amino acid starvation resulting in the phosphorylation of GCN2 and eIF2α in *Shigella flexneri* and *Salmonella typhimurium* infection (Tattoli et al., 2012). This was also shown to promote the formation of SGs in both organisms. The same group found that HRI was also activated in *S. flexneri* infection, and upon knockdown of HRI, the number of cells containing SGs decreased (Abdel-Nour et al., 2019). A similar effect occurred with knockdown of GCN2, while a double knockdown reduced both P-eIF2α below detection as well as eliminating SGs from cells. Furthermore, these kinases were found to have distinct roles, with HRI alone required for inflammatory responses from the ISR.

*Pseudomonas aeruginosa* most likely increases the P-eIF2α in infected cells by multiple kinases (as described above in the PERK section). Other kinases that were implicated in elevated P-eIF2α during infection include HRI and GCN2. Elevated P-eIF2α caused downstream protein expression (GADD34 and CHOP) in infected cells, which was tapered by the knockdown of HRI (van ‘t Wout et al., 2015). A possible mechanism was proposed to be iron depletion. GCN2 and PERK may also be activated by *P. aeruginosa.* One study determined that GCN2 activation occurred dependent on pyocyanin, a *P. aeruginosa* virulence factor (Yang et al., 2016). In addition, as pyocyanin can cause ROS generation (Hall et al., 2016), it is possible that the PERK pathway may also be activated, although this was not examined (Yang et al., 2016).

## Can Antibiotics Alter Stress Granule Biology?

The dynamics of SGs can be manipulated by drugs and other small molecules, leading to their induction, modification, or disassembly (Wang et al., 2020). Among these, several drugs used for cancer treatment have been shown to impact SGs. One example of such an effect are drugs that cause SG assembly through increased P-eIF2α; these include fluorouracil, etoposide, and cisplatin (García et al., 2011; Kaehler et al., 2014; Vilas-Boas et al., 2016).

Much less is known about the effect of antimicrobial antibiotics on SGs. One example of a clear effect on SGs is the antibiotic puromycin. It is an aminonucleoside antibiotic that has long been used in the study of SGs, due to its ability to promote SG disassembly through premature peptide chain termination and the resulting destabilization of polysomes (Kedersha et al., 2000). Similarly, the antibiotic translation inhibitor anisomycin, which is used as an anti-fungal and anti-protozoan agent, inhibits SG assembly in arsenite-stressed cells (Fang et al., 2019). Interestingly, this study also found that in the absence of external stress, anisomycin increased P-eIF2α without inducing SG assembly. The effect of anisomycin is not unexpected, as it is thought to stall eukaryotic translation similarly to cycloheximide, a common drug used in SG analyses (Chan et al., 2004).

Since antibiotics that target prokaryotic translation do not generally affect eukaryotic translation, they would not be expected to induce SGs by altering translation. However, neomycin, an aminoglycoside prokaryotic protein synthesis inhibitor, reduced the assembly of SGs following an external stress through an unknown mechanism (Fang et al., 2019). This is consistent with the ability of some aminoglycosides to alter eukaryotic translation (Prokhorova et al., 2017). In contrast, another aminoglycoside, kanamycin, was shown to induce SG formation in mouse hair cells (Gonçalves et al., 2019). Its mechanism of action on SG assembly is unclear due to it being 250-fold less active against eukaryotic translation *in vitro* than prokaryotic ribosomes (McCoy et al., 2013).

While serendipity can reveal interactions between antibiotics and SG biology, screens have demonstrated that many small molecules impact SGs (Christen et al., 2019; Fang et al., 2019; Wheeler et al., 2019). One of these studies revealed that psammaplysin F, an bactericidal antibiotic produced from a marine sponge, reduced the number of SGs following exposure to sodium arsenite, through decreased P-eIF2α (Christen et al., 2019). Interestingly, approximately 100 small molecules were identified in another screen of two cell lines where SGs were induced by arsenite, including neomycin, as discussed above (Fang et al., 2019). Unsurprisingly, the majority of the antibiotics affecting SGs in this study were those targeting eukaryotic cells, such as fungi and protozoa, including mebendazole, fenbendazole, and dapsone. One example that stood out in this study was the antibiotic gramicidin S. Gramicidin S targets Gram-positive bacteria and fungi and was found to result in fewer SGs and exhibited diffused SG protein fluorescence throughout the cytoplasm. Gramicidin acts to form pores that only allow passage of monovalent cations. The differences and similarities to other bacterial pore-forming toxins would be of interest for further exploration.

Generally speaking, it is clear that some antibiotics can affect SGs. Due to their selectivity for microbes, especially those targeting bacteria, it could be expected that interactions affecting SGs would result from altered mechanisms of action in the host cell. It remains to be investigated to what extent they can exert their effect and the ultimate consequences of their function.

## What Is the Impact of Stress Granules on Microbial Infection?

Many roles have been ascribed for SGs during viral infection, which have been studied for well over a decade (Eiermann et al., 2020). However, it is clear that infection with many non-viral microbes can also induce SG formation. Potential roles for SGs in the host can be to limit infection and affect intracellular signaling. Recent results suggest two possible manners by which SGs can impact infection. First, the ISR can be activated, elevating P-eIF2α, which inhibits translation and promotes the assembly of SGs. Second, more recent studies suggest an important linkage between innate immunity and SGs.

Studies have not examined the role of SGs *per se* in inhibition of infection. However, there is clear evidence that P-eIF2α and the upstream activating kinases can affect infections. What remains unclear is whether these effects are due to the translation inhibition caused by P-eIF2α, or if they are linked to the formation of SGs themselves.

### The Integrated Stress Response Can Affect the Outcome of Microbial Infection

Most studies in this regard have examined activation of the eIF2α kinase PERK. Drug-induced activation of PERK inhibited microbial infection in several manners. *Campylobacter jejuni* was shown to be particularly inhibited by PERK activation (Tentaku et al., 2018). Addition of the drugs thapsigargin and tunicamycin reduced the ability for the bacteria to invade Caco-2 cells. Inducing PERK signaling by pre-infection with the bacteria also decreased *C. jejuni* invasion suggesting an antibacterial role of PERK activation. Finally, knockdown of the three main UPR-signaling proteins (PERK, ATF6, and IRE1) facilitated bacterial infection in HeLa cells. A similar effect was observed in *Listeria*, where activation of PERK with tunicamycin and thapsigargin decreased recovery of intracellular bacteria (Pillich et al., 2012).

Another mechanism by which ISR could operate to combat infection is by avoidance of host cell apoptosis as exemplified by *Mycobacterium bovis* (Seimon et al., 2010). Apoptosis of macrophages is thought to be an important host defense against infection. However, infection of murine peritoneal macrophages with this bacteria at low MOI does not cause apoptosis (Seimon et al., 2010). When combined with PERK activation by thapsigargin, there was a significant, up to fivefold synergistic increase in apoptosis. Intriguingly, it has been suggested that virulent *Mycobacterium* strains have mechanisms to avoid apoptosis and promote survival, of which activation of PERK could be a component (Lam et al., 2017). Thus, inhibition of PERK, P-eIF2α, or SGs could be a generalized mechanism to promote mycobacterial virulence.

In contrast, PERK activation appears beneficial for at least three microbes. First, infection with the intracellular bacteria *Brucella melitensis* is reduced by inhibition of PERK activation by pre-treatment with tauroursodeoxycholic acid (Smith et al., 2013). This reduced *Brucella* CFU recovery from RAW cells by at least 10-fold 24 hpi. Second, the protozoan parasite *P. berghei* also appears to benefit from PERK activation by tunicamycin (Inácio et al., 2015). Mice that were intraperitoneally treated with tunicamycin and injected with the parasite had a significant increase in liver infection compared with mice without the drug. Finally, the intracellular bacteria *Chlamydia muridarum* appears to benefit from UPR activation including PERK activation, both *in vitro* and *in vivo* (George et al., 2017). However, this may not implicate SGs, as similar reductions in recovery of inclusion-forming units were found in drugs targeting PERK as well as IRE1, which is another arm of the UPR. Nevertheless, these studies together are suggestive that at least for a subset of organisms, depending on the lifestyle, PERK, P-eIF2α, and/or SGs could promote virulence.

Protein kinase R was also shown to affect microbial infection. PKR decreased parasite killing through autophagy in *Toxoplasma gondii* (Ogolla et al., 2013; Portillo et al., 2017). Dominant negative PKR reduced protozoan killing and, in knockout mice, resulted in a greater number of infected mice.

HRI activation is important in combatting *Listeria* infection (Bahnan et al., 2018). Replication of *Listeria* increased in macrophages lacking HRI. This was replicated *in vivo*, where knockout mice were more susceptible to listeriosis. This was further supported by treatment of mice with the P-eIF2α activator I-17. The elevated P-eIF2α resulted in a reduced recovery of CFU from peritoneal exudate macrophages.

Finally, expression of the S51A mutant of eIF2α, which cannot be phosphorylated, promotes infection. *Chlamydia* and *Listeria* infection results in a greater than fivefold increase in inclusion forming units from knock-in mutant MEF cells, with *Yersinia* infection exhibiting a 25-fold increase (Shrestha et al., 2012).

## Stress Granule Relationship With the Innate Immune Response

There are well-established connections between SGs, the innate immune response, and viral infection (Cláudio et al., 2013; Onomoto et al., 2014; McCormick and Khaperskyy, 2017). In contrast, connections between these processes during microbial infection are just beginning to be identified.

In the last few years, new work has shown a convergence between innate immunity pathways and SGs. Two examples are the cytosolic DNA sensor cyclic GMP-AMP synthase (cGAS) and inflammasomes, which activate inflammatory responses including pyroptosis, a lytic form of cell death caused as a result of intracellular infection.

Cyclic GMP-AMP synthase is an important cytoplasmic DNA sensor for detection of microbial or self-DNA. The core SG protein G3BP1 has been shown to associate with cGAS and be critical for its production of type I IFN (Liu et al., 2019). This study suggested that cGAS was not related to SG formation since cGAS was not found in SGs. In addition, knockdown of TIA1, which eliminated SGs, did not affect cGAS signaling. Furthermore, cytoplasmic cGAS foci induced with interferon stimulatory DNA did not colocalize with G3BP1.

However, the extent of SGs’ role in cGAS activation remains unclear. A subsequent study supported the finding that G3BP1 regulated cGAS (Hu et al., 2019). In contrast to the previous work, the researchers found that the cGAS foci contained G3BP1, PKR, and P-eIF2α. That these foci are SGs is further supported by interferon stimulatory DNA-induced PKR activation and elevated P-eIF2α, dependent on cGAS; however, it remains to be confirmed whether these G3BP1 foci are *bona fide* SGs.

The NLRP3 inflammasome appears to be more directly affected or regulated by SGs. While SGs promote survival, inflammasomes promote pyroptosis. The DEAD box helicase DDX3X can interact and regulate the NLRP3 inflammasome (Samir et al., 2019). Overexpression of DDX3X has been previously shown to cause SG formation and is impaired when downregulated, although it is likely not involved in SG disassembly (Shih et al., 2011; Cui et al., 2020). The interaction of DDX3X with the inflammasome combines with its role in SG biology as documented in Samir et al. (2019), which demonstrated that SG assembly can prevent the activation of inflammasomes, while pre-assembled inflammasomes are unaffected by SG formation. In contrast to cGAS however, G3BP1 depletion did not affect the function of inflammasomes. This suggests that different immune pathways may be differently affected by SGs and SG proteins.

An interesting connection between innate immunity and SGs is the ability of eIF2α kinases to regulate the signalosomes, which have been shown to have the ability to form amyloid fibrils (Sohn and Hur, 2016; Girardin et al., 2020). These include the NLRP3 and AIM2 inflammasome, the nodosome, as well as MAVS, TRIF, and RIPK1/RIPK3 amyloid-like fibrils. There is also a connection between innate immunity and amyloid-driven neurodegeneration (Ryu et al., 2018). SGs have numerous links to both neurodegeneration and alternative structures promoted by prion-like proteins (Wolozin and Ivanov, 2019). These connections suggest that infection may result in a variety of effects dependent on SGs and phase separation of signaling proteins.

## Future Perspectives

In many ways, the current state of SG research in non-viral microbial infection is similar to research into its role during viral infection in the early 2000s (Anderson and Kedersha, 2002). At that time, evidence suggested SG formation as a common response to viral infection, especially as elevated P-eIF2α levels and eIF2α kinase activation had been observed during infection. In the nearly two decades since, the extent of the interactions between viruses and SGs have become both clear and extensive (Eiermann et al., 2020). This includes the adaptations in which viruses subvert SGs and their protein components.

Stress granules, and liquid–liquid phase-separated membraneless organelles more generally have been proposed to have multiple roles in the cell. Several of these functions can be important for the host during infection. These include serving as a reaction crucible by concentrating factors within SGs, as an organizational hub (such as seen in the nucleolus and in chromosomes) as well as sequestration of signaling complexes to coordinate responses to stresses including infection (Shin and Brangwynne, 2017).

Currently, these multiple functions remain unexplored. While three bacterial species have been shown to form SGs in infected cells, the studies revealing their existence emphasized the role of the ISR, rather than SGs *per se* (Tattoli et al., 2012; Vonaesch et al., 2016; Abdel-Nour et al., 2019). SGs were more linked to a consequence of the ISR rather than the active participants in the host cell defense against bacterial infection. However, further study is needed to understand if SGs themselves can affect infection and its outcomes.

Interestingly, the area in microbial infection biology where SGs functions have been examined most explicitly is the innate immune response (Hu et al., 2019; Liu et al., 2019; Samir et al., 2019). These results are likely to be extended as SGs become more widely studied as a response to multiple microbial infections.

For example, inflammasomes are important in the innate immune response to bacterial infection (von Moltke et al., 2013). SGs appear to regulate whether they assemble in response to infection mediated by sequestration of the DEAD box helicase DDX3X (Samir et al., 2019). With further study of SGs, this process may be generalizable to many microbial infections. Potential evidence of SGs affecting inflammasomes could be occurring in *S. pneumoniae*. Pneumococcal H_2_O_2_ has been independently shown to inhibit inflammasome assembly and greatly increase P-eIF2α levels, likely sufficient for SG assembly (Loose et al., 2015; Erttmann and Gekara, 2019).

While SGs have not been examined for specific roles outside of innate immune responses, the upstream activators, as components of the ISR, reveal them as a potentially important element in host defenses against infection. These studies have demonstrated that inhibition of the ISR often positively impacts the infectious organisms in areas such as increased replication. These data also reveal that some intracellular microbes have co-opted this response to promote infection.

In addition, SGs have been shown to be impacted by additional biological processes, which are relevant during microbial infection; however these are beyond the scope of this review. These processes include autophagy, the mTOR signaling pathway, and proteasomal function, among others. First, since autophagy was shown to be important for SG clearing, there has been much work linking the two pathways (Buchan et al., 2013; Abildgaard et al., 2020). Second, mTOR, phosphoinositide 3-kinase (PI3K), and the p38 mitogen-activated protein kinases have been shown to impact SG assembly and infection, making these kinases an interesting avenue for further research (Martin et al., 2012; Fournier et al., 2013; Sfakianos et al., 2018; Heberle et al., 2019). Finally, a linkage has been found between the ubiquitin–proteasome system and both SG assembly and microbial infection (Mazroui et al., 2007; Lin and Machner, 2017).

In this review, we have outlined studies in which SGs have been identified during non-viral microbial infection. We argue that host SG assembly is a widespread reaction to microbial infection. The ISR, that is elevated P-eIF2α and eIF2α kinase activation, appears to be a general response to microbial infections. As a result of ISR activation, these infections can form SGs. Further investigation into the role of SGs during infection should reveal important antimicrobial functions imparted by SGs. Analogous to viral infections, these studies also reveal exceptions to ISR activation and suggest that microbes may also have the ability to not only inhibit SG formation but also assist in their disassembly. This suggests that microbial infections, like viral infections, may provoke adaptations to modulate the SG response.

## Author Contributions

AT and TN wrote the manuscript, made substantial, direct and intellectual contribution to the work, and approved it for publication. Both authors contributed to the article and approved the submitted version.

## Conflict of Interest

The authors declare that the research was conducted in the absence of any commercial or financial relationships that could be construed as a potential conflict of interest.

## References

[B1] Abdel-Nour M. Carneiro L. A. M. Downey J. Tsalikis J. Outlioua A. Prescott D. (2019). The heme-regulated inhibitor is a cytosolic sensor of protein misfolding that controls innate immune signaling. *Science* 365:eaaw4144. 10.1126/science.aaw4144 31273097 PMC10433729

[B2] Abildgaard M. H. Brynjólfsdóttir S. H. Frankel L. B. (2020). The autophagy-RNA interplay: degradation and beyond. *Trends Biochem. Sci.* 45 845–857. 10.1016/j.tibs.2020.07.007 32828649

[B3] Adomavicius T. Guaita M. Zhou Y. Jennings M. D. Latif Z. Roseman A. M. (2019). The structural basis of translational control by eIF2 phosphorylation. *Nat. Commun.* 10:2136. 10.1038/s41467-019-10167-3 31086188 PMC6513899

[B4] Anand S. S. Babu P. P. (2013). Endoplasmic reticulum stress and neurodegeneration in experimental cerebral malaria. *Neurosignals* 21 99–111. 10.1159/000336970 22584375

[B5] Anderson P. Kedersha N. (2002). Stressful initiations. *J. Cell Sci.* 115:3227. 10.1242/jcs.115.16.3227 12140254

[B6] Bahnan W. Boucher J. C. Gayle P. Shrestha N. Rosen M. Aktas B. (2018). The eIF2α Kinase Heme-regulated inhibitor protects the host from infection by regulating intracellular pathogen trafficking. *Infect. Immun.* 86:e0707-17. 10.1128/IAI.00707-17 29311243 PMC5820965

[B7] Besic V. Habibolahi F. Noël B. Rupp S. Genovesio A. Lebreton A. (2020). Coordination of transcriptional and translational regulations in human epithelial cells infected by *Listeria monocytogenes*. *RNA Biol.* 17 1492–1507. 10.1080/15476286.2020.1777380 32584699 PMC7549700

[B8] Böhme L. Albrecht M. Riede O. Rudel T. (2010). *Chlamydia trachomatis*-infected host cells resist dsRNA-induced apoptosis. *Cell. Microbiol.* 12 1340–1351. 10.1111/j.1462-5822.2010.01473.x 20482554

[B9] Bounedjah O. Hamon L. Savarin P. Desforges B. Curmi P. A. Pastré D. (2012). Macromolecular crowding regulates assembly of mRNA stress granules after osmotic stress: new role for compatible osmolytes. *J. Biol. Chem.* 287 2446–2458. 10.1074/jbc.M111.292748 22147700 PMC3268405

[B10] Brostrom C. O. Brostrom M. A. (1997). “Regulation of translational initiation during cellular responses to stress,” in *Progress in Nucleic Acid Research and Molecular Biology*, ed. Moldave K. (Cambridge, MA: Academic Press), 79–125. 10.1016/S0079-6603(08)60034-3 9308364

[B11] Buchan J. R. Kolaitis R.-M. Taylor J. P. Parker R. (2013). Eukaryotic stress granules are cleared by autophagy and Cdc48/VCP Function. *Cell* 153 1461–1474. 10.1016/j.cell.2013.05.037 23791177 PMC3760148

[B12] Burwick N. Aktas B. H. (2017). The eIF2-alpha kinase HRI: a potential target beyond the red blood cell. *Expert Opin. Ther. Targets* 21 1171–1177. 10.1080/14728222.2017.1397133 29063813 PMC5761058

[B13] Celli J. Tsolis R. M. (2015). Bacteria, the endoplasmic reticulum and the unfolded protein response: friends or foes? *Nat. Rev. Microbiol.* 13 71–82. 10.1038/nrmicro3393 25534809 PMC4447104

[B14] Chan J. Khan S. N. Harvey I. Merrick W. Pelletier J. (2004). Eukaryotic protein synthesis inhibitors identified by comparison of cytotoxicity profiles. *RNA* 10 528–543. 10.1261/rna.5200204 14970397 PMC1370947

[B15] Choi H.-H. Shin D.-M. Kang G. Kim K.-H. Park J. B. Hur G. M. (2010). Endoplasmic reticulum stress response is involved in *Mycobacterium tuberculosis* protein ESAT-6-mediated apoptosis. *FEBS Lett.* 584 2445–2454. 10.1016/j.febslet.2010.04.050 20416295

[B16] Choi J.-A. Lim Y.-J. Cho S.-N. Lee J.-H. Jeong J. A. Kim E. J. (2013). Mycobacterial HBHA induces endoplasmic reticulum stress-mediated apoptosis through the generation of reactive oxygen species and cytosolic Ca2+ in murine macrophage RAW 264.7 cells. *Cell Death Dis.* 4:e957. 10.1038/cddis.2013.489 24336077 PMC3877560

[B17] Christen K. E. Davis R. A. Kennedy D. (2019). Psammaplysin F increases the efficacy of bortezomib and sorafenib through regulation of stress granule formation. *Int. J. Biochem. Cell Biol.* 112 24–38. 10.1016/j.biocel.2019.04.008 31022461

[B18] Cláudio N. Dalet A. Gatti E. Pierre P. (2013). Mapping the crossroads of immune activation and cellular stress response pathways. *EMBO J.* 32 1214–1224. 10.1038/emboj.2013.80 23584529 PMC3642686

[B19] Coburn B. Sekirov I. Finlay B. B. (2007). Type III secretion systems and disease. *Clin. Microbiol. Rev.* 20:535. 10.1128/CMR.00013-07 17934073 PMC2176049

[B20] Cui B. C. Sikirzhytski V. Aksenova M. Lucius M. D. Levon G. H. Mack Z. T. (2020). Pharmacological inhibition of DEAD-Box RNA Helicase 3 attenuates stress granule assembly. *Biochem. Pharmacol.* 182:114280. 10.1016/j.bcp.2020.114280 33049245 PMC7686075

[B21] de Jong M. F. Starr T. Winter M. G. den Hartigh A. B. Child R. Knodler L. A. (2013). Sensing of bacterial Type IV secretion via the unfolded protein response. *mBio* 4:e0418-12. 10.1128/mBio.00418-12 23422410 PMC3624511

[B22] Dias-Teixeira K. L. Calegari-Silva T. C. Medina J. M. Vivarini ÁC. Cavalcanti Á Teteo N. (2017). Emerging role for the PERK/eIF2α/ATF4 in Human Cutaneous *Leishmaniasis*. *Sci. Rep.* 7:17074. 10.1038/s41598-017-17252-x 29213084 PMC5719050

[B23] Eiermann N. Haneke K. Sun Z. Stoecklin G. Ruggieri A. (2020). Dance with the devil: stress granules and signaling in antiviral responses. *Viruses* 12:984. 10.3390/v12090984 32899736 PMC7552005

[B24] English B. C. Van Prooyen N. Örd T. Örd T. Sil A. (2017). The transcription factor CHOP, an effector of the integrated stress response, is required for host sensitivity to the fungal intracellular pathogen *Histoplasma capsulatum*. *PLoS Pathog.* 13:e1006589. 10.1371/journal.ppat.1006589 28953979 PMC5633207

[B25] Erttmann S. F. Gekara N. O. (2019). Hydrogen peroxide release by bacteria suppresses inflammasome-dependent innate immunity. *Nat. Commun.* 10:3493. 10.1038/s41467-019-11169-x 31375698 PMC6677825

[B26] Fang M. Y. Markmiller S. Vu A. Q. Javaherian A. Dowdle W. E. Jolivet P. (2019). Small-molecule modulation of TDP-43 recruitment to stress granules prevents persistent TDP-43 accumulation in ALS/FTD. *Neuron* 103 802–819.e11. 10.1016/j.neuron.2019.05.048 31272829 PMC6728177

[B27] Fournier M.-J. Coudert L. Mellaoui S. Adjibade P. Gareau C. Côté M.-F. (2013). Inactivation of the mTORC1-eukaryotic translation initiation factor 4E pathway alters stress granule formation. *Mol. Cell. Biol.* 33:2285. 10.1128/MCB.01517-12 23547259 PMC3648080

[B28] Frädrich C. Beer L.-A. Gerhard R. (2016). Reactive oxygen species as additional determinants for Cytotoxicity of *Clostridium difficile* Toxins A and B. *Toxins* 8:25. 10.3390/toxins8010025 PMC4728547 26797634

[B29] García M. A. Carrasco E. Aguilera M. Alvarez P. Rivas C. Campos J. M. (2011). The chemotherapeutic Drug 5-fluorouracil promotes PKR-mediated apoptosis in a p53- independent manner in colon and breast cancer cells. *PLoS One* 6:e23887. 10.1371/journal.pone.0023887 21887339 PMC3161074

[B30] García M. A. Gil J. Ventoso I. Guerra S. Domingo E. Rivas C. (2006). Impact of protein kinase PKR in cell biology: from antiviral to antiproliferative action. *Microbiol. Mol. Biol. Rev.* 70 1032–1060. 10.1128/MMBR.00027-06 17158706 PMC1698511

[B31] Gekara N. O. Groebe L. Viegas N. Weiss S. (2008). *Listeria monocytogenes* desensitizes immune cells to subsequent Ca2+ signaling via Listeriolysin O-induced depletion of intracellular Ca2+ stores. *Infect. Immun.* 76:857. 10.1128/IAI.00622-07 18056478 PMC2223449

[B32] George Z. Omosun Y. Azenabor A. A. Goldstein J. Partin J. Joseph K. (2019). The molecular mechanism of induction of unfolded protein response by Chlamydia. *Biochem. Biophys. Res. Commun.* 508 421–429. 10.1016/j.bbrc.2018.11.034 30503337 PMC6343654

[B33] George Z. Omosun Y. Azenabor A. A. Partin J. Joseph K. Ellerson D. (2017). The Roles of unfolded protein response pathways in chlamydia pathogenesis. *J. Infect. Dis.* 215 456–465. 10.1093/infdis/jiw569 27932618 PMC6455037

[B34] Girardin S. E. Cuziol C. Philpott D. J. Arnoult D. (2020). The eIF2α kinase HRI in innate immunity, proteostasis, and mitochondrial stress. *FEBS J.* 10.1111/febs.15553. [Epub ahead of print]. 32892501

[B35] Go D. Lee J. Choi J.-A. Cho S.-N. Kim S.-H. Son S.-H. (2019). Reactive oxygen species-mediated endoplasmic reticulum stress response induces apoptosis of *Mycobacterium avium*-infected macrophages by activating regulated IRE1-dependent decay pathway. *Cell. Microbiol.* 21:e13094. 10.1111/cmi.13094 31386788 PMC6899680

[B36] Gonçalves A. C. Towers E. R. Haq N. Porco J. A. Pelletier J. Dawson S. J. (2019). Drug-induced stress granule formation protects sensory hair cells in mouse cochlear explants during Ototoxicity. *Sci. Rep.* 9:12501. 10.1038/s41598-019-48393-w 31467369 PMC6715625

[B37] Gonzalez M. R. Bischofberger M. Frêche B. Ho S. Parton R. G. van der Goot F. G. (2011). Pore-forming toxins induce multiple cellular responses promoting survival. *Cell. Microbiol.* 13 1026–1043. 10.1111/j.1462-5822.2011.01600.x 21518219

[B38] Grabiner M. A. Fu Z. Wu T. Barry K. C. Schwarzer C. Machen T. E. (2014). *Pseudomonas aeruginosa* quorum-sensing molecule homoserine lactone modulates inflammatory signaling through PERK and eI-F2α. *J. Immunol.* 193:1459. 10.4049/jimmunol.1303437 24990083 PMC4113911

[B39] Grohmann E. Christie P. J. Waksman G. Backert S. (2018). Type IV secretion in Gram-negative and Gram-positive bacteria. *Mol. Microbiol.* 107 455–471. 10.1111/mmi.13896 29235173 PMC5796862

[B40] Grönberg A. Zettergren L. Bergh K. Ståhle M. Heilborn J. Ängeby K. (2010). Antioxidants protect Keratinocytes against *M. ulcerans* mycolactone Cytotoxicity. *PLoS One* 5:e13839. 10.1371/journal.pone.0013839 21079804 PMC2973957

[B41] Guillén-Boixet J. Kopach A. Holehouse A. S. Wittmann S. Jahnel M. Schlüßler R. (2020). RNA-induced conformational switching and clustering of G3BP drive stress granule assembly by condensation. *Cell* 181 346–361.e17. 10.1016/j.cell.2020.03.049 32302572 PMC7181197

[B42] Hall S. McDermott C. Anoopkumar-Dukie S. McFarland A. Forbes A. Perkins A. V. (2016). Cellular effects of Pyocyanin, a secreted virulence factor of *Pseudomonas aeruginosa*. *Toxins* 8:236. 10.3390/toxins8080236 PMC4999852 27517959

[B43] Hanada H. Ikeda-Dantsuji Y. Naito M. Nagayama A. (2003). Infection of human fibroblast-like synovial cells with *Chlamydia trachomatis* results in persistent infection and interleukin-6 production. *Microb. Pathog.* 34 57–63. 10.1016/S0882-4010(02)00189-4 12623273

[B44] Hanson K. K. Mair G. R. (2014). Stress granules and *Plasmodium* liver stage infection. *Biol. Open* 3:103. 10.1242/bio.20136833 24357231 PMC3892165

[B45] Heberle A. M. Razquin Navas P. Langelaar-Makkinje M. Kasack K. Sadik A. Faessler E. (2019). The PI3K and MAPK/p38 pathways control stress granule assembly in a hierarchical manner. *Life Sci. Alliance* 2:e201800257. 10.26508/lsa.201800257 30923191 PMC6441495

[B46] Hershey J. W. B. (1989). Protein phosphorylation controls translation rates. *J. Biol. Chem.* 264 20823–20826. 2687263

[B47] Hofmann S. Kedersha N. Anderson P. Ivanov P. (2021). Molecular mechanisms of stress granule assembly and disassembly. *Biochim. Biophys. Acta Mol. Cell Res.* 1868:118876. 10.1016/j.bbamcr.2020.118876 33007331 PMC7769147

[B48] Hu L. Raybourne R. B. Kopecko D. J. (2005). Ca2+ release from host intracellular stores and related signal transduction during *Campylobacter jejuni* 81-176 internalization into human intestinal cells. *Microbiology* 151 3097–3105. 16151220 10.1099/mic.0.27866-0

[B49] Hu S. Sun H. Yin L. Li J. Mei S. Xu F. (2019). PKR-dependent cytosolic cGAS foci are necessary for intracellular DNA sensing. *Sci. Signal.* 12:eaav7934. 10.1126/scisignal.aav7934 31772125

[B50] Ietta F. Maioli E. Daveri E. Gonzaga Oliveira J. da Silva R. J. Romagnoli R. (2017). Rottlerin-mediated inhibition of *Toxoplasma gondii* growth in BeWo trophoblast-like cells. *Sci. Rep.* 7:1279. 10.1038/s41598-017-01525-6 28455500 PMC5430667

[B51] Inácio P. Zuzarte-Luís V. Ruivo M. T. Falkard B. Nagaraj N. Rooijers K. (2015). Parasite-induced ER stress response in hepatocytes facilitates *Plasmodium* liver stage infection. *EMBO Rep.* 16 955–964. 10.15252/embr.201439979 26113366 PMC4552488

[B52] Jalihal A. P. Pitchiaya S. Xiao L. Bawa P. Jiang X. Bedi K. (2020). Multivalent proteins rapidly and reversibly phase-separate upon osmotic cell volume change. *Mol. Cell* 79 978–990.e5. 10.1016/j.molcel.2020.08.004 32857953 PMC7502480

[B53] Kaehler C. Isensee J. Hucho T. Lehrach H. Krobitsch S. (2014). 5-Fluorouracil affects assembly of stress granules based on RNA incorporation. *Nucleic Acids Res.* 42 6436–6447. 10.1093/nar/gku264 24728989 PMC4041438

[B54] Kedersha N. Cho M. R. Li W. Yacono P. W. Chen S. Gilks N. (2000). Dynamic shuttling of Tia-1 accompanies the recruitment of mRNA to mammalian stress granules. *J. Cell Biol.* 151 1257–1268. 10.1083/jcb.151.6.1257 11121440 PMC2190599

[B55] Kiehn T. E. Edwards F. F. Brannon P. Tsang A. Y. Maio M. Gold J. W. (1985). Infections caused by *Mycobacterium avium* complex in immunocompromised patients: diagnosis by blood culture and fecal examination, antimicrobial susceptibility tests, and morphological and seroagglutination characteristics. *J. Clin. Microbiol.* 21:168. 10.1128/jcm.21.2.168-173.1985 PMC271607 3972985

[B56] Kloft N. Neukirch C. Bobkiewicz W. Veerachato G. Busch T. von Hoven G. (2010). Pro-autophagic signal induction by bacterial pore-forming toxins. *Med. Microbiol. Immunol.* 199 299–309. 10.1007/s00430-010-0163-0 20454906 PMC2955911

[B57] Kopp M. C. Larburu N. Durairaj V. Adams C. J. Ali M. M. U. (2019). UPR proteins IRE1 and PERK switch BiP from chaperone to ER stress sensor. *Nat. Struct. Mol. Biol.* 26 1053–1062. 10.1038/s41594-019-0324-9 31695187 PMC6858872

[B58] Lam A. Prabhu R. Gross C. M. Riesenberg L. A. Singh V. Aggarwal S. (2017). Role of apoptosis and autophagy in tuberculosis. *Am. J. Physiol. Lung Cell Mol. Physiol.* 313 L218–L229. 10.1152/ajplung.00162.2017 28495854 PMC5582934

[B59] Lee S.-Y. Lee M.-S. Cherla R. P. Tesh V. L. (2008). Shiga toxin 1 induces apoptosis through the endoplasmic reticulum stress response in human monocytic cells. *Cell. Microbiol.* 10 770–780. 10.1111/j.1462-5822.2007.01083.x 18005243

[B60] Lim Y.-J. Choi J.-A. Choi H.-H. Cho S.-N. Kim H.-J. Jo E.-K. (2011). Endoplasmic reticulum stress pathway-mediated apoptosis in macrophages contributes to the survival of *Mycobacterium tuberculosis*. *PLoS One* 6:e28531. 10.1371/journal.pone.0028531 22194844 PMC3237454

[B61] Lim Y.-J. Choi J.-A. Lee J.-H. Choi C. H. Kim H.-J. Song C.-H. (2015). *Mycobacterium tuberculosis* 38-kDa antigen induces endoplasmic reticulum stress-mediated apoptosis via toll-like receptor 2/4. *Apoptosis* 20 358–370. 10.1007/s10495-014-1080-2 25544271

[B62] Lin Y.-H. Machner M. P. (2017). Exploitation of the host cell ubiquitin machinery by microbial effector proteins. *J. Cell Sci.* 130:1985. 10.1242/jcs.188482 28476939 PMC5482977

[B63] Liu Z.-S. Cai H. Xue W. Wang M. Xia T. Li W.-J. (2019). G3BP1 promotes DNA binding and activation of cGAS. *Nat. Immunol.* 20 18–28. 10.1038/s41590-018-0262-4 30510222 PMC8276115

[B64] Lloyd R. E. (2012). How do viruses interact with stress-associated RNA granules? *PLoS Pathog.* 8:e1002741. 10.1371/journal.ppat.1002741 22761570 PMC3386173

[B65] Loose M. Hudel M. Zimmer K.-P. Garcia E. Hammerschmidt S. Lucas R. (2015). Pneumococcal hydrogen peroxide-induced stress signaling regulates inflammatory genes. *J. Infect. Dis.* 211 306–316. 10.1093/infdis/jiu428 25183769 PMC4334831

[B66] Martin S. Saha B. Riley J. L. (2012). The battle over mTOR: an emerging theatre in host-pathogen immunity. *PLoS Pathog.* 8:e1002894. 10.1371/journal.ppat.1002894 23028309 PMC3441621

[B67] Masson G. R. (2019). Towards a model of GCN2 activation. *Biochem. Soc. Trans.* 47 1481–1488. 10.1042/BST20190331 31647517 PMC6824675

[B68] Mazroui R. Di Marco S. Kaufman R. J. Gallouzi I.-E. (2007). Inhibition of the ubiquitin-proteasome system induces stress granule formation. *Mol. Biol. Cell* 18 2603–2618. 10.1091/mbc.e06-12-1079 17475769 PMC1924830

[B69] McCormick C. Khaperskyy D. A. (2017). Translation inhibition and stress granules in the antiviral immune response. *Nat. Rev. Immunol.* 17 647–660. 10.1038/nri.2017.63 28669985

[B70] McCoy L. S. Roberts K. D. Nation R. L. Thompson P. E. Velkov T. Li J. (2013). Polymyxins and analogues bind to ribosomal RNA and interfere with eukaryotic translation in vitro. *Chembiochem* 14 2083–2086. 10.1002/cbic.201300496 24105917 PMC3947458

[B71] Mehlitz A. Karunakaran K. Herweg J.-A. Krohne G. van de Linde S. Rieck E. (2014). The chlamydial organism *Simkania negevensis* forms ER vacuole contact sites and inhibits ER-stress. *Cell. Microbiol.* 16 1224–1243. 10.1111/cmi.12278 24528559

[B72] Morinaga N. Yahiro K. Matsuura G. Moss J. Noda M. (2008). Subtilase cytotoxin, produced by Shiga-toxigenic *Escherichia coli*, transiently inhibits protein synthesis of Vero cells via degradation of BiP and induces cell cycle arrest at G1 by downregulation of cyclin D1. *Cell. Microbiol.* 10 921–929. 10.1111/j.1462-5822.2007.01094.x 18005237 PMC3021990

[B73] Moulder J. W. (1991). Interaction of chlamydiae and host cells in vitro. *Microbiol. Rev.* 55:143. 10.1128/mr.55.1.143-190.1991 PMC372804 2030670

[B74] Mpiga P. Ravaoarinoro M. (2006). *Chlamydia trachomatis* persistence: an update. *Microbiol. Res.* 161 9–19. 10.1016/j.micres.2005.04.004 16338585

[B75] Ogbechi J. Hall B. S. Sbarrato T. Taunton J. Willis A. E. Wek R. C. (2018). Inhibition of Sec61-dependent translocation by mycolactone uncouples the integrated stress response from ER stress, driving cytotoxicity via translational activation of ATF4. *Cell Death Dis.* 9:397. 10.1038/s41419-018-0427-y 29540678 PMC5852046

[B76] Ogolla P. S. Portillo J.-A. C. White C. L. Patel K. Lamb B. Sen G. C. (2013). The Protein Kinase double-stranded RNA-dependent (PKR) enhances protection against disease cause by a non-viral pathogen. *PLoS Pathog.* 9:e1003557. 10.1371/journal.ppat.1003557 23990781 PMC3749959

[B77] Ohmer M. Tzivelekidis T. Niedenführ N. Volceanov-Hahn L. Barth S. Vier J. (2019). Infection of HeLa cells with *Chlamydia trachomatis* inhibits protein synthesis and causes multiple changes to host cell pathways. *Cell. Microbiol.* 21:e12993. 10.1111/cmi.12993 30551267

[B78] Onomoto K. Yoneyama M. Fung G. Kato H. Fujita T. (2014). Antiviral innate immunity and stress granule responses. *Trends Immunol.* 35 420–428. 10.1016/j.it.2014.07.006 25153707 PMC7185371

[B79] Pakos-Zebrucka K. Koryga I. Mnich K. Ljujic M. Samali A. Gorman A. M. (2016). The integrated stress response. *EMBO Rep.* 17 1374–1395. 10.15252/embr.201642195 27629041 PMC5048378

[B80] Pan Q. Wang X. Liu T. Yu Y. Li L. Zhou R. (2020). *Mycoplasma hyopneumoniae* inhibits Porcine Beta-Defensin 2 production by blocking the unfolded protein response to facilitate epithelial adhesion and infection. *Infect. Immun.* 88:e0164-20. 10.1128/IAI.00164-20 32312764 PMC7309629

[B81] Pereira R. M. S. Dias Teixeira K. L. Barreto-de-Souza V. Calegari-Silva T. C. De-Melo L. D. B. Soares D. C. (2010). Novel role for the double-stranded RNA-activated protein kinase PKR: modulation of macrophage infection by the protozoan parasite *Leishmania*. *FASEB J.* 24 617–626. 10.1096/fj.09-140053 19812373

[B82] Pillich H. Loose M. Zimmer K.-P. Chakraborty T. (2012). Activation of the unfolded protein response by *Listeria monocytogenes*. *Cell. Microbiol.* 14 949–964. 10.1111/j.1462-5822.2012.01769.x 22321539

[B83] Portillo J.-A. C. Muniz-Feliciano L. Lopez Corcino Y. Lee S. J. Van Grol J. Parsons S. J. (2017). *Toxoplasma gondii* induces FAK-Src-STAT3 signaling during infection of host cells that prevents parasite targeting by autophagy. *PLoS Pathog.* 13:e1006671. 10.1371/journal.ppat.1006671 29036202 PMC5658194

[B84] Prokhorova I. Altman R. B. Djumagulov M. Shrestha J. P. Urzhumtsev A. Ferguson A. (2017). Aminoglycoside interactions and impacts on the eukaryotic ribosome. *Proc. Natl. Acad. Sci. U.S.A.* 114:E10899. 10.1073/pnas.1715501114 29208708 PMC5754804

[B85] Repp H. Pamukçi Z. Koschinski A. Domann E. Darji A. Birringer J. (2002). Listeriolysin of *Listeria monocytogenes* forms Ca2+-permeable pores leading to intracellular Ca2+ oscillations. *Cell. Microbiol.* 4 483–491. 10.1046/j.1462-5822.2002.00207.x 12174083

[B86] Riggs C. L. Kedersha N. Ivanov P. Anderson P. (2020). Mammalian stress granules and P bodies at a glance. *J. Cell Sci.* 133:jcs242487. 10.1242/jcs.242487 32873715 PMC10679417

[B87] Ruggieri A. Dazert E. Metz P. Hofmann S. Bergeest J.-P. Mazur J. (2012). Dynamic oscillation of translation and stress granule formation mark the cellular response to virus infection. *Cell Host Microb.* 12 71–85. 10.1016/j.chom.2012.05.013 22817989 PMC3873964

[B88] Ryu J. K. Rafalski V. A. Meyer-Franke A. Adams R. A. Poda S. B. Rios Coronado P. E. (2018). Fibrin-targeting immunotherapy protects against neuroinflammation and neurodegeneration. *Nat. Immunol.* 19 1212–1223. 10.1038/s41590-018-0232-x 30323343 PMC6317891

[B89] Sadighi Akha A. A. Theriot C. M. Erb-Downward J. R. McDermott A. J. Falkowski N. R. Tyra H. M. (2013). Acute infection of mice with *Clostridium difficile* leads to eIF2α phosphorylation and pro-survival signalling as part of the mucosal inflammatory response. *Immunology* 140 111–122. 10.1111/imm.12122 23668260 PMC3809711

[B90] Samir P. Kesavardhana S. Patmore D. M. Gingras S. Malireddi R. K. S. Karki R. (2019). DDX3X acts as a live-or-die checkpoint in stressed cells by regulating NLRP3 inflammasome. *Nature* 573 590–594. 10.1038/s41586-019-1551-2 31511697 PMC6980284

[B91] Sanders D. W. Kedersha N. Lee D. S. W. Strom A. R. Drake V. Riback J. A. (2020). Competing Protein-RNA interaction networks control multiphase intracellular organization. *Cell* 181 306–324.e28. 10.1016/j.cell.2020.03.050 32302570 PMC7816278

[B92] Schröder M. (2008). Endoplasmic reticulum stress responses. *Cell. Mole. Life Sci.* 65 862–894. 10.1007/s00018-007-7383-5 18038217 PMC11131897

[B93] Seimon T. A. Kim M.-J. Blumenthal A. Koo J. Ehrt S. Wainwright H. (2010). Induction of ER stress in macrophages of *Tuberculosis granulomas*. *PLoS One* 5:e12772. 10.1371/journal.pone.0012772 20856677 PMC2939897

[B94] Sfakianos A. P. Mellor L. E. Pang Y. F. Kritsiligkou P. Needs H. Abou-Hamdan H. (2018). The mTOR-S6 kinase pathway promotes stress granule assembly. *Cell Death Differ.* 25 1766–1780. 10.1038/s41418-018-0076-9 29523872 PMC6004310

[B95] Shih J.-W. Wang W.-T. Tsai T.-Y. Kuo C.-Y. Li H.-K. Wu Lee Y.-H. (2011). Critical roles of RNA helicase DDX3 and its interactions with eIF4E/PABP1 in stress granule assembly and stress response. *Biochem. J.* 441 119–129. 10.1042/BJ20110739 21883093

[B96] Shima K. Klinger M. Schütze S. Kaufhold I. Solbach W. Reiling N. (2015). The role of endoplasmic reticulum-related BiP/GRP78 in interferon gamma-induced persistent *Chlamydia pneumoniae* infection. *Cell. Microbiol.* 17 923–934. 10.1111/cmi.12416 25588955

[B97] Shin Y. Brangwynne C. P. (2017). Liquid phase condensation in cell physiology and disease. *Science* 357:eaaf4382. 10.1126/science.aaf4382 28935776

[B98] Shrestha N. Bahnan W. Wiley D. J. Barber G. Fields K. A. Schesser K. (2012). Eukaryotic Initiation Factor 2 (eIF2) signaling regulates proinflammatory cytokine expression and bacterial invasion. *J. Biol. Chem.* 287 28738–28744. 10.1074/jbc.M112.375915 22761422 PMC3436510

[B99] Smith J. A. Khan M. Magnani D. D. Harms J. S. Durward M. Radhakrishnan G. K. (2013). Brucella induces an unfolded protein response via TcpB that supports intracellular replication in macrophages. *PLoS Pathog.* 9:e1003785. 10.1371/journal.ppat.1003785 24339776 PMC3855547

[B100] Sohn J. Hur S. (2016). Filament assemblies in foreign nucleic acid sensors. *Curr. Opin. Struc. Biol.* 37 134–144. 10.1016/j.sbi.2016.01.011 26859869 PMC5070476

[B101] Song E. J. Lee S.-J. Lim H. S. Kim J. S. Jang K. K. Choi S. H. (2016). *Vibrio vulnificus* VvhA induces autophagy-related cell death through the lipid raft-dependent c-Src/NOX signaling pathway. *Sci. Rep.* 6:27080. 10.1038/srep27080 27250250 PMC4890043

[B102] Sun C. Wang H. Chen S. Li Z. Li S. Wang J. (2014). Recombinant *Clostridium difficile* toxin B induces endoplasmic reticulum stress in mouse colonal carcinoma cells. *Acta Biochim. Biophys. Sin.* 46 973–981. 10.1093/abbs/gmu091 25274332

[B103] Tattoli I. Sorbara M. T. Vuckovic D. Ling A. Soares F. Carneiro L. A. M. (2012). Amino acid starvation induced by invasive bacterial pathogens triggers an innate host defense program. *Cell Host Microb.* 11 563–575. 10.1016/j.chom.2012.04.012 22704617

[B104] Tattoli I. Sorbara M. T. Yang C. Tooze S. A. Philpott D. J. Girardin S. E. (2013). Listeria phospholipases subvert host autophagic defenses by stalling pre-autophagosomal structures. *EMBO J.* 32 3066–3078. 10.1038/emboj.2013.234 24162724 PMC3844955

[B105] Tentaku A. Shimohata T. Hatayama S. Kido J. Nguyen A. Q. Kanda Y. (2018). Host cellular unfolded protein response signaling regulates *Campylobacter jejuni* invasion. *PLoS One* 13:e0205865. 10.1371/journal.pone.0205865 30321237 PMC6188877

[B106] van ‘t Wout E. F. A. van Schadewijk A. van Boxtel R. Dalton L. E. Clarke H. J. Tommassen J. (2015). Virulence factors of *Pseudomonas aeruginosa* induce both the unfolded protein and integrated stress responses in airway epithelial cells88. *PLoS Pathog.* 11:e1004946. 10.1371/journal.ppat.1004946 26083346 PMC4471080

[B107] Vilas-Boas F. A. D. S. da Silva A. M. de Sousa L. P. Lima K. M. Vago J. P. Bittencourt L. F. F. (2016). Impairment of stress granule assembly via inhibition of the eIF2alpha phosphorylation sensitizes glioma cells to chemotherapeutic agents. *J. Neurooncol.* 127 253–260. 10.1007/s11060-015-2043-3 26732083

[B108] von Hoven G. Kloft N. Neukirch C. Ebinger S. Bobkiewicz W. Weis S. (2012). Modulation of translation and induction of autophagy by bacterial exoproducts. *Med. Microbiol. Immunol.* 201 409–418. 10.1007/s00430-012-0271-0 22991039 PMC3470817

[B109] von Moltke J. Ayres J. S. Kofoed E. M. Chavarría-Smith J. Vance R. E. (2013). Recognition of bacteria by inflammasomes. *Annu. Rev. Immunol.* 31 73–106. 10.1146/annurev-immunol-032712-095944 23215645

[B110] Vonaesch P. Campbell-Valois F.-X. Dufour A. Sansonetti P. J. Schnupf P. (2016). *Shigella* flexneri modulates stress granule composition and inhibits stress granule aggregation. *Cell. Microbiol.* 18 982–997. 10.1111/cmi.12561 27282465

[B111] Walter P. Ron D. (2011). The unfolded protein response: from stress pathway to homeostatic regulation. *Science* 334:1081. 10.1126/science.1209038 22116877

[B112] Wang F. Li J. Fan S. Jin Z. Huang C. (2020). Targeting stress granules: a novel therapeutic strategy for human diseases. *Pharmacol. Res.* 161:105143. 10.1016/j.phrs.2020.105143 32814168 PMC7428673

[B113] Webster S. J. Ellis L. O’Brien L. M. Tyrrell B. Fitzmaurice T. J. Elder M. J. (2016). IRE1α mediates PKR activation in response to *Chlamydia trachomatis* infection. *Microb. Infect.* 18 472–483. 10.1016/j.micinf.2016.03.010 27021640 PMC4936793

[B114] Wek R. C. (2018). Role of eIF2α kinases in translational control and adaptation to cellular stress. *Cold Spring Harb. Perspect. Biol.* 10:a032870. 10.1101/cshperspect.a032870 29440070 PMC6028073

[B115] Wen Y. Luo F. Zhao Y. Su S. Shu M. Li Z. (2020). *Chlamydia trachomatis* plasmid-encoded protein pORF5 activates unfolded protein response to induce autophagy via MAPK/ERK signaling pathway. *Biochem. Biophys. Res. Commun.* 527 805–810. 10.1016/j.bbrc.2020.04.117 32446560

[B116] Wheeler R. J. Lee H. O. Poser I. Pal A. Doeleman T. Kishigami S. (2019). Small molecules for modulating protein driven liquid-liquid phase separation in treating neurodegenerative disease. *bioRxiv* [Preprint]. 10.1101/721001

[B117] White J. P. Lloyd R. E. (2012). Regulation of stress granules in virus systems. *Trends Microbiol.* 20 175–183. 10.1016/j.tim.2012.02.001 22405519 PMC3322245

[B118] Wiley D. J. Shrestha N. Yang J. Atis N. Dayton K. Schesser K. (2009). The activities of the Yersinia protein kinase A (YpkA) and outer protein J (YopJ) virulence factors converge on an eIF2alpha kinase. *J. Biol. Chem.* 284 24744–24753. 10.1074/jbc.M109.010140 19553678 PMC2757178

[B119] Wolfson J. J. May K. L. Thorpe C. M. Jandhyala D. M. Paton J. C. Paton A. W. (2008). Subtilase cytotoxin activates PERK, IRE1 and ATF6 endoplasmic reticulum stress-signalling pathways. *Cell. Microbiol.* 10 1775–1786. 10.1111/j.1462-5822.2008.01164.x 18433465 PMC2575110

[B120] Wolozin B. Ivanov P. (2019). Stress granules and neurodegeneration. *Nat. Rev. Neurosci.* 20 649–666. 10.1038/s41583-019-0222-5 31582840 PMC6986315

[B121] Yang P. Mathieu C. Kolaitis R.-M. Zhang P. Messing J. Yurtsever U. (2020). G3BP1 is a tunable switch that triggers phase separation to assemble stress granules. *Cell* 181 325–345.e28. 10.1016/j.cell.2020.03.046 32302571 PMC7448383

[B122] Yang Z.-S. Ma L.-Q. Zhu K. Yan J.-Y. Bian L. Zhang K.-Q. (2016). *Pseudomonas* toxin pyocyanin triggers autophagy: Implications for pathoadaptive mutations. *Autophagy* 12 1015–1028. 10.1080/15548627.2016.1170256 27159636 PMC4922443

[B123] Zhang K. Kaufman R. J. (2008). From endoplasmic-reticulum stress to the inflammatory response. *Nature* 454 455–462. 10.1038/nature07203 18650916 PMC2727659

